# High-end intestinal ultrasound versus mid-end systems benchmarked against tandem ileocolonoscopy in inflammatory bowel disease (HUMID): a paired prospective, validating confirmatory study

**DOI:** 10.1016/j.eclinm.2026.103856

**Published:** 2026-04-01

**Authors:** Partha Pal, Mohammad Abdul Mateen, Syed Misba Naaz, Kanapuram Pooja, Radhika Nittala, Ayush Singh, Bharath Kumar Reddy, Praveen Kumar Reddy Vasepally, Sayyed Mahiboob Najbuddin, Tummalapalli Satya Maharshi, Uday Kumar Marri, Zaheer Nabi, Palle Manohar Reddy, Jahangeer Basha, Rajesh Gupta, Manu Tandan, Rupjyoti Talukdar, D. Nageshwar Reddy

**Affiliations:** a Medical Gastroenterology, Asian Institute of Gastroenterology, India; b Diagnostic Radiology and Ultrasound, Asian Institute of Gastroenterology, India; c Interventional Radiology, Asian Institute of Gastroenterology, India

**Keywords:** Intestinal ultrasound, Crohn's disease, Ulcerative colitis, Colonoscopy, Inflammatory bowel disease

## Abstract

**Background:**

Intestinal ultrasound (IUS) is redefining inflammatory bowel disease (IBD) monitoring, but global adoption remains limited by the cost of high-end systems. This study evaluated whether widely available mid-range equipment provides comparable diagnostic accuracy without additional cost.

**Methods:**

We conducted a prospective, cross-sectional, paired diagnostic accuracy study at a single IBD centre in India from September 2024 to October 2025. We included patients aged 18–75 years with confirmed ulcerative colitis (UC) or Crohn's disease (CD). Trained operators performed same-day blinded assessments using a mid-end (Siemens ACUSON S2000) and a high-end system (Samsung RS80 EVO). Blinded ileocolonoscopy with central review served as the reference. Endoscopic remission was defined as Ulcerative Colitis Endoscopic Index of Severity (UCEIS) ≤ 1 for UC and Simple Endoscopic Score for Crohn's Disease (SES-CD) ≤ 2 for CD. Sonographic activity was assessed using the Milan Ultrasound Criteria (MUC) for UC and the International Bowel Ultrasound Simplified Activity Score (IBUS-SAS) for CD. Diagnostic accuracy, segment-wise performance, and reclassification impact were evaluated. Paired comparisons used McNemar testing for proportions, receiver operating characteristic (ROC) analysis with area-under-the-curve (AUC) comparison, and agreement analyses using Bland–Altman and concordance methods. This study is registered with ClinicalTrials.gov, NCT06938295.

**Findings:**

450 patients underwent paired IUS and ileocolonoscopy (239 UC, 211 CD; median age 36 years; 32% female). In UC, sensitivity was 94.2% with the mid-end and 97.1% with the high-end system (Δ +2.9%; 95% CI –3.8 to 9.6), with identical specificity (54.5%). In CD, sensitivity was 85.1% versus 87.6% (Δ +2.5%; 95% CI –8.2 to 13.0) and specificity 62.0% versus 60.0% (95% CI –28.0 to 24.2); accuracies differed by <3%. Segment-wise performance showed no significant differences across any bowel regions. ROC analyses showed good–excellent discrimination [AUC: 0.75–0.93 (UC); 0.83–0.94 (CD) in various segments], with overlapping curves and strong score correlation. Reclassification analysis showed a net gain of +6 patients (diagnostic odds ratio 1.8, 95% CI: 0.9–3.5) for high end machine. Bland–Altman and concordance analyses demonstrated minimal bias and high agreement. Decision-curve analysis confirmed equivalent net clinical benefit.

**Interpretation:**

High-end IUS conferred no measurable diagnostic or clinical advantage over mid-range equipment when examinations were performed by trained operators using standardised scoring systems, supporting mid-range systems as scalable and cost-effective tools for IBD monitoring.

**Funding:**

No external funding.

**Research in context**

**Evidence before this study**

We searched PubMed, Embase, and Web of Science from Jan 1, 1990, to Aug 31, 2025, without language restrictions, using combinations of the terms “intestinal ultrasound”, “bowel ultrasound”, “inflammatory bowel disease”, “ulcerative colitis”, “Crohn's disease”, “diagnostic accuracy”, “monitoring”, and “endoscopy”, and screened reference lists of relevant systematic reviews, consensus statements, and guidelines. Most studies have focused on intestinal ultrasound as a technique rather than on the performance of different ultrasound hardware platforms. Existing evidence comparing systems is limited to indirect comparisons, small explanatory studies, or evaluations of hand-held devices against conventional machines. A recent large, prospective, real-world study demonstrated that intestinal ultrasound performed using existing mid-range ultrasound equipment can accurately guide clinical decision-making and frequently obviate colonoscopy and cross-sectional imaging, but this study did not include a direct, paired comparison with high-end platforms. Consequently, whether higher-cost, high-end ultrasound systems confer a meaningful diagnostic or clinical advantage over widely available mid-range systems has remained uncertain.

**Added value of this study**

This study provides the first large, prospective, same-patient, same-day comparison of mid-range and high-end intestinal ultrasound systems, benchmarked against blinded ileocolonoscopy. In a real-world cohort of patients with ulcerative colitis and Crohn's disease, mid-range ultrasound systems matched high-end platforms across all clinically relevant domains: sensitivity and specificity for active inflammation, segment-wise diagnostic performance, discrimination of endoscopic activity, detection of complications, quantitative agreement of bowel wall thickness and validated activity scores, and net clinical benefit for treatment decisions. This study directly demonstrates that higher-cost ultrasound technology does not confer incremental diagnostic or therapeutic advantage in inflammatory bowel disease. These findings shift the evidence base from proof-of-concept to proof-of-equivalence and redefine the determinants of high-quality intestinal ultrasound as training, standardisation, and workflow integration rather than device tier.

**Implications of all the available evidence**

Taken together with existing evidence, these findings indicate that the clinical accuracy and utility of intestinal ultrasound are determined predominantly by operator training, standardised acquisition, and validated scoring frameworks rather than by ultrasound hardware tier. Widely available mid-range ultrasound systems can therefore be used with confidence for treat-to-target monitoring, assessment of therapeutic response, escalation or de-escalation of therapy, and detection of complications in inflammatory bowel disease, without compromising diagnostic performance or clinical decision-making. At a health-system level, this evidence supports a shift in implementation strategy away from investment in high-cost imaging platforms towards scalable training programmes and structured service models, enabling equitable expansion of intestinal ultrasound in low- and middle-income countries and in resource-constrained healthcare settings.

## Introduction

Intestinal ultrasound (IUS) is transforming IBD care by enabling real-time, radiation-free assessment of bowel inflammation, treatment response, and complications. Validated against endoscopy and cross-sectional imaging, IUS is now central to treat to target strategies recommended by international guidelines.^1^, ^2^, ^3^, ^4^ High-end ultrasound platforms have improved image quality and accelerated global interest in IUS; however, their cost limits widespread adoption, particularly in emerging and resource-constrained settings.^5^, ^6^, ^7^

Mid-range ultrasound systems, which are already widely available in routine clinical practice, may offer a scalable alternative if shown to provide comparable diagnostic performance. Early prospective data from our centre demonstrated that such mid-range systems can accurately assess disease activity, independently influence management and reduced the need for colonoscopy or cross-sectional imaging in ∼50% patients, supporting the feasibility of a lower-cost IUS service.^8^, ^9^, ^10^ However, whether these systems perform comparably to high-end platforms with advanced processing power and bowel presets remains unknown. Recent prospective evidence also indicates that even hand-held ultrasound devices can deliver assessments comparable to conventional systems, further suggesting that hardware tier may be less critical than technique and training.^11^ No head-to-head comparison has yet clarified whether superior hardware meaningfully improves diagnostic accuracy or outcomes, or if operator expertise and standardisation are the true determinants of IUS precision.

The HUMID (*h*igh-end *u*ltrasound versus *m*id-end ultrasound with tandem *i*leo-colonoscopy in inflammatory bowel *d*isease) study was designed to address this evidence gap through a rigorously paired, prospective comparison of mid- and high-end ultrasound platforms, operated by IUS trainers at an internationally certified IUS training centre blinded to each other's findings. Using blinded ileocolonoscopy with central review as the reference standard, the study evaluated diagnostic concordance, segment-wise accuracy, and the clinical impact of system-specific findings. By determining whether the diagnostic performance of mid-range IUS is comparable to that of high-end systems, the HUMID study aims to define the clinical and economic rationale for scalable global deployment of intestinal ultrasound.

## Methods

### Study design and participants

The HUMID study was a prospective, paired, head-to-head diagnostic accuracy study conducted at the Asian Institute of Gastroenterology (AIG), Hyderabad, India, a high-volume tertiary IBD centre with an internationally certified training programme in intestinal ultrasound (IUS). The study compared the diagnostic accuracy of a high-end ultrasound system (Samsung RS80 EVO) and a mid-end system (Siemens ACUSON S2000) for assessing ileocolonic inflammation, using blinded ileocolonoscopy as the reference standard. The protocol was approved by the institutional ethics committee (AIG/IEC-BH&R 61/09.2024-01) and conducted from September 2024 to October 2025 in accordance with the Declaration of Helsinki. All participants provided written informed consent, and the study was registered at ClinicalTrials.gov (NCT06938295). Patients or the public were not involved in the design, conduct, or reporting of the study.

Consecutive adults aged 18–75 years with confirmed CD or UC affecting the colon and/or terminal ileum were screened in outpatient and inpatient settings. Patients were enrolled when ileocolonoscopy was clinically warranted for suspected flare, therapeutic response assessment, or monitoring of quiescent disease. Each participant first underwent a mid-end IUS examination followed by a blinded high-end IUS examination; ileocolonoscopy was then performed when indicated. Exclusion criteria were contraindication to colonoscopy, pregnancy, age <18 years or >75 years, inability to provide consent, and postoperative CD or UC, as surgically altered anatomy precluded reliable segmental correlation between ultrasound and ileocolonoscopy. Patients with isolated proximal small-bowel CD beyond the terminal ileum were not included, as ileocolonoscopy was used as the uniform reference standard and does not allow reliable segmental correlation in these regions.

### Procedures

At the study centre, all patients undergo structured clinical assessment using the Harvey–Bradshaw Index (HBI) for CD and the Simple Clinical Colitis Activity Index (SCCAI) for UC before IUS by clinical research associates (K.P., S.M.N.). In this study, after providing informed consent, consecutive patients underwent a mid-end IUS examination using the Siemens ACUSON S2000 (Siemens Healthcare, Erlangen, Germany) performed by an experienced gastrointestinal radiologist (M.A.M.). Each patient was then independently re-examined using a high-end Samsung RS80 EVO system (Samsung India Electronics Pvt. Ltd, Haryana, India) by a gastroenterologist trained and certified in IUS (P.P.), who remained blinded to the mid-end findings. Ileocolonoscopy was subsequently performed when clinically indicated according to routine care pathways, in which the high-end IUS assessment contributed to decision-making. High-end ultrasound systems differ from mid-range platforms by offering greater computational capacity (often quantified in billion operations per second), single-crystal transducer technology, broader frequency bandwidth, enhanced image-processing algorithms, improved depth penetration and resolution uniformity, dedicated bowel presets, and substantially higher capital cost (see Supplementary Material page 1 and Supplementary Table S1 for details).^8^

Both operators were highly experienced IUS practitioners (>1500 examinations jointly), served as faculty at an International Bowel Ultrasound (IBUS)-certified training centre, and co-authored multiple IUS studies.^4^^,^^8^^,^^12^^,^^13^ Before study initiation, they performed twenty pilot benchmarking scans (10 each UC and CD) in each system to harmonise acquisition settings, measurement techniques, and reporting standards across platforms. These examinations were independently saved and re-measured, with discrepancies resolved by consensus by another radiologist (U.K.M). Inter-observer agreement during pilot benchmarking was assessed in representative segments using intraclass correlation coefficients (ICC), demonstrating excellent reliability (ICC 0.96 for ileal wall thickness in CD and 0.94 for sigmoid wall thickness in UC). Throughout both the pilot benchmarking phase and the main study, each operator performed examinations exclusively on their routinely assigned platform (M.A.M. on the mid-end system and P.P. on the high-end system), reflecting established institutional workflow and ensuring consistency with real-world clinical practice.

A fixed scan order (mid-end followed by high-end) was used for all participants. Mid-end examinations were performed using a 4–9 MHz linear probe (9L4, frequency ≥7 MHz), with paediatric abdominal and thyroid presets selected in the absence of a dedicated bowel preset as described in our prior prospective study (Supplementary Figure S1).^8^ High-end examinations used 2–9 MHz and 2–18 MHz linear probes with single-crystal technology. Each scan assessed bowel wall thickness (BWT), layer stratification, vascularity, inflammatory fat, lymphadenopathy, and complications (stricture, fistula, abscess). Active disease was defined as BWT >3 mm (>4 mm for rectum on transperineal ultrasound-TPUS) and/or hypervascularity (modified Limberg ≥1, assessed at flow 5–8 cm/s), loss of stratification, or inflammatory fat (Supplement Page 1).^13^^,^^14^ BWT was calculated as the mean of two longitudinal and two transverse measurements. All segments were documented systematically with still images and cine loops in accordance with European Crohn's and Colitis Organisation (ECCO) and European Society of Gastrointestinal and Abdominal Radiology (ESGAR) recommendations (Supplement Page 2 and 3).^15^ Sonographic activity was quantified using the Milan Ultrasound Criteria (MUC) for UC and the International Bowel Ultrasound Segmental Activity Score (IBUS-SAS) for CD.^16^

Ileo-colonoscopy (using CF-H180AL/I, Olympus Medical Systems, Tokyo, Japan) was performed on the same or following day when clinically indicated, after 2 L polyethylene glycol preparation under propofol sedation. Experienced endoscopists (>2000 colonoscopies and >500 IBD colonoscopy) (A.S., B.K.R., P.K.R.V., S.M.N.) blinded to all ultrasound findings graded disease using the Simple Endoscopic Score for Crohn's Disease (SES-CD) and the Ulcerative Colitis Endoscopic Index of Severity (UCEIS); active disease was defined as SES-CD ≥3 or UCEIS ≥2. All procedures were video-recorded and centrally reviewed by a blinded IBD expert (R.N.) to minimise variability in endoscopic scoring.

### Outcomes

The primary outcome of this study was to compare the diagnostic accuracy of IUS using a high-end versus a mid-end system for detecting ileocolonic inflammation, using ileocolonoscopy as the reference standard. For each system, sensitivity, specificity, positive predictive value (PPV), negative predictive value (NPV), and overall accuracy were calculated (see Supplementary Table S2 for glossary of terms).

The secondary outcome was the impact of high-end IUS on clinical management decisions, quantified as the proportion of patients whose treatment plan changed and whether these changes represented correct or incorrect reclassification relative to ileocolonoscopy. Management decisions were adjudicated through a structured, blinded review by three independent panels of experienced gastroenterologists: one panel (R.G. , P.M.R.) assessed management based on mid-end IUS findings, a second panel (J.B., Z.N.) reassessed management incorporating high-end IUS findings, and a third panel (M.T., D. N. R.), blinded to both ultrasound assessments, used ileocolonoscopy to determine the reference disease state. Management impact was summarised using the net reclassification improvement (NRI) and the management diagnostic odds ratio (DOR).

Exploratory analyses included:

(1) Segment-wise concordance between systems and against endoscopy, with segment definitions harmonised to validated endoscopic scoring systems. For CD, caecum and ascending colon were combined as the right colon and descending and sigmoid colon as the left colon; the IUS segment with highest activity was used when multiple segments mapped to a single endoscopic region. For UC, ileal IUS measurements were excluded because the UCEIS does not quantitatively assess the ileum.
(2) Correlation analyses between sonographic activity indices (MUC, IBUS-SAS) and endoscopic scores (UCEIS, SES-CD).
(3) Discriminative performance for predicting endoscopic activity, assessed using receiver operating characteristic (ROC) curves and area under the curve (AUC).
(4) Agreement and interchangeability analyses
(5) Net clinical benefit of each system across a range of threshold probabilities

### Statistical analysis

Categorical variables were summarised as frequencies and percentages, and continuous variables as medians with ranges. Analyses were performed separately for ulcerative colitis (UC) and Crohn's disease (CD). The primary diagnostic comparison evaluated the absolute difference in accuracy metrics—sensitivity, specificity, positive predictive value (PPV), negative predictive value (NPV), and overall accuracy—between high-end and mid-end IUS, using ileocolonoscopy as the reference standard. Absolute differences with 95% confidence intervals (CIs) were calculated for each diagnostic metric using standard formulae for paired proportions, and McNemar's test was used for paired comparisons. As this was a paired within-patient comparison, treatment exposure was identical for both ultrasound systems at the time of assessment and was therefore not included as a covariate in primary analyses.

Segment-wise exploratory analyses applied the same paired framework. Correlation analyses assessed associations between sonographic indices (BWT, MUC, IBUS-SAS, vascularity) and endoscopic activity (UCEIS for UC, SES-CD for CD) using Spearman's rank correlation coefficient (ρ). Normality of continuous variables was assessed using the Shapiro–Wilk test. Agreement and interchangeability between systems were evaluated using Bland–Altman plots (mean difference and limits of agreement), concordance correlation coefficients (CCC), and calibration analyses (slope and intercept from linear regression of high-end versus mid-end measurements).

Clinical acceptability of bowel wall thickness was pre-specified as an inter-system difference ≤1 mm, and acceptability proportions were calculated for each segment. Decision curve analysis (DCA) was performed to estimate the net clinical benefit of ultrasound-based logistic regression models across threshold probabilities from 0.05 to 0.95, relative to “treat-all” and “treat-none” strategies. For these models, the overall MUC score (ulcerative colitis) or IBUS-SAS score (Crohn's disease) obtained from each ultrasound system was used as the predictor, and endoscopically active disease served as the binary outcome (reference standard).

Inter- or intra-observer reliability statistics (ICC, κ) were not calculated because each ultrasound system was operated by a single experienced ultrasonographer; therefore, the study was designed primarily to compare platform performance under routine workflow conditions rather than to estimate observer reproducibility.

Sample size estimation used a paired McNemar design. The calculation was based on our previous prospective mid-end IUS study, which demonstrated an overall diagnostic accuracy of 84.9% versus ileocolonoscopy (sensitivity 80.3%, specificity 93.8%).^8^ Assuming a 10% absolute improvement with the high-end system and using α = 0.05 and 80% power, approximately 150 paired participants were required for a single comparison.^17^ Because UC and CD were analysed independently, and to ensure adequate precision for secondary and segment-wise analyses, the prespecified target sample size was ∼400 participants. All analyses were two-sided (α = 0.05) and performed using SPSS v26 (Armonk, NY, USA). Python version 3.11 was used for DCA and related visualisation, employing NumPy and pandas for data handling and Matplotlib for plotting.

### Role of the funding source

No external funding supported the HUMID study. All high-end and mid-end intestinal ultrasound examinations were performed as part of routine clinical workflow at no additional cost to patients. Ileo-colonoscopy was undertaken when clinically indicated as standard care; for participants requiring only sigmoidoscopy, a full ileo-colonoscopy was performed without extra cost as a part of this research protocol.

## Results

### Patient and baseline characteristics

**Fig. 1:**
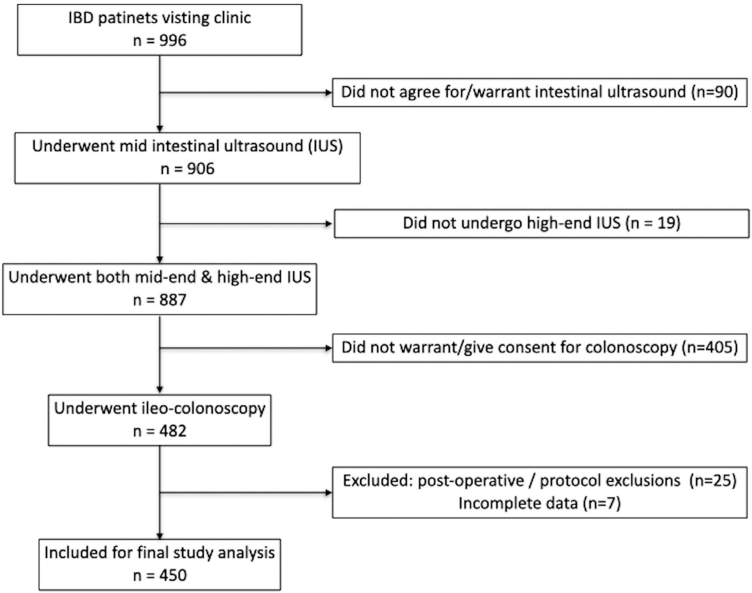
Study profile. Illustrates the recruitment and diagnostic workflow of the study.

During the study period, 996 patients with established IBD attended the clinic; 887 underwent paired mid-end and high-end IUS, and 482 proceeded to ileo-colonoscopy. After exclusions, 450 patients were included (Fig. 1): 239 (53.1%) with ulcerative colitis (UC) and 211 (46.8%) with Crohn's disease (CD). The median age was 36 years (range 18–75), and one-third were female. In UC, most patients had left-sided (53.6%) or extensive colitis (27.6%), while CD was predominantly ileal (35.1%) or ileocolonic (37.4%) in location. CD patients mainly exhibited inflammatory behaviour (81.9%), with fewer stricturing (13.3%) or penetrating (4.8%) phenotypes and perianal disease in 6.1%. Clinical indications reflected routine practice, with over half evaluated during quiescent disease and the remainder for flare assessment or treatment-response monitoring. Treatment patterns differed by disease: 5-ASA and tofacitinib were common in UC, whereas azathioprine and biologics were more widely used in CD. Full baseline characteristics are presented in Table 1. Segmental bowel wall thickness, MUC, IBUS-SAS, and corresponding endoscopic activity indices showed overlapping distributions between systems, as detailed in Supplementary Tables S3 and S4.

**Table 1:** Baseline characteristics of patients with ulcerative colitis and Crohn's disease.

Characteristic	Ulcerative colitis (n = 239)	Crohn's disease (n = 211)
Number of patients	239 (100.0)	211 (100.0)
Age, years—median (range)	36 (18–69)	35 (18–75)
Gender—Female (%)	66 (27.6)	78 (37)
Smoker-current/former (%)	9 (3.8)	14 (6.5)
Duration of disease in months, median (range)	21 (4–252)	18 (3–240)
Indication—Clinically quiescent disease (%)	149 (62.5)	119 (56.4)
Indication—Assessing response on therapy	48 (20.1)	60 (28.4)
Indication—Flare (%)	42 (17.6)	32 (15.2)
Clinical activity scores	Simple clinical colitis activity index, median (range): 3 (0–14)	Harvey Bradshaw Index, median (range): 4 (0–18)
Extent, n (%)	E1 (Proctitis): 45 (18.8) E2 (Prostosigmoiditis): 128 (53.6) E3 (Extensive colitis): 66 (27.6)	L1 (terminal ileal): 74 (35.1) L2 (colonic): 45 (21.3) L3 (ileocolonic): 79 (37.4) L4 (Proximal small bowel) + L1/L3: 11 (5.3)
Behaviour	B1 (inflammatory) 240 (100.0)	B1 (inflammatory) 173 (81.9) B2 (stricturing) 28 (13.3) B3 (fistulizing) 10 (4.8) p (perianal disease) 13 (6.1)
5-ASA use, n (%)	191 (79.9)	78 (37)
Azathioprine use, n (%)	74 (31)	115 (54.5)
Methotrexate use, n (%)	–	10 (4.7%)
Systemic steroid use, n (%)	9 (3.8)	17 (8.1)
Budesonide use, n (%)	15 (6.3)	15 (7.1)
Infliximab use, n (%)	–	10 (4.7)
Adalimumab use, n (%)	–	21 (10)
Vedolizumab use, n (%)	1 (0.4)	1 (0.5)
Tofacitinib use, n (%)	31 (13)	1 (0.5)
Ustekinumab use, n (%)	0 (0.0)	1 (0.5)
Upadacitinib use, n (%)	0 (0.0)	6 (2.8)

### IUS findings and diagnostic accuracy

Across both UC and CD, high-end and mid-end intestinal ultrasound yielded high diagnostic performance, with no statistically significant superiority of either platform. In UC, sensitivity was 97.1% with the high-end system and 94.2% with the mid-end system (Δ +2.9%; 95% CI –3.8 to +9.6; p = 0.125), while specificity was identical at 54.5% (Δ 0%; 95% CI –25 to +25). In CD, sensitivity was 87.6% versus 85.1% (Δ +2.5%; 95% CI –8.2 to +13; p = 0.20) and specificity 60.0% versus 62.0% (Δ −2.0%; 95% CI –28 to +24.2; p = 0.70). Overall accuracy differed by less than 3% in both diseases, with rare and symmetric discordant classifications, indicating absence of systematic bias (Tables 2 and 3). Segment-level analyses demonstrated similar pattern of equivalence. No bowel segment showed a statistically significant absolute difference between systems. In CD, the terminal ileum and right colon showed numerically higher sensitivity for the high-end system, but the absolute differences were not statistically significant. The transverse and left colon demonstrated uniformly high specificity (>94%) and nearly identical sensitivity between platforms in both diseases (Tables 2 and 3). In CD, complication detection showed identical specificity (100%) for both systems, with a small, numerical difference in sensitivity between systems (Δ +10%; 95% CI –6.3 to +45.9; p = 0.16) (Table 3). In 28 patients with UC and 31 with CD, missed disease corresponded to mild endoscopic inflammation or aphthous ulceration without associated transmural thickening.

**Table 2:** Overall and segment-wise diagnostic performance comparing mid-end and high-end intestinal ultrasound systems in ulcerative colitis across the whole colon and individual segments.

Metric	Mid-end	High-end	Δ (High–Mid)	Discordant (b,c)	p-value
Overall analysis					
Sensitivity	94.2%	97.1%	+2.9% (−3.8 to +9.6)	b1 = 1, c1 = 6	0.125
Specificity	54.5%	54.5%	0% (−25 to +25)	b2 = 9, c2 = 9	1.00
PPV	84.5%	84.8%	+0.3% (−6.8 to +7.5)	–	–
NPV	78.3%	87.8%	+9.5% (−6 to +25.1)	–	–
Accuracy	83.3%	85.4%	+2.1% (−4.4 to +8.6)	–	–

Metric (rectum)	Mid-end	High-end	Δ (High–Mid)	Discordant pairs	p-value (exact)
Rectum segment analysis					
Sensitivity	95.5%	96.8%	+1.3% (−1.2 to +3.8)	b1 = 0, c1 = 1	1.00
Specificity	61.0%	53.7%	−7.3% (−17.5 to +2.9)	b2 = 13, c2 = 7	0.26
PPV	82.4%	80.0%	−2.4% (−10.3% to +5.5%)	–	–
NPV	87.7%	89.8%	+2.1% (−9.9 to +14.1)	–	–
Accuracy	83.7%	82.0%	−1.7% (−5.4 to +2.1)		0.19

Metric (sigmoid)	Mid-end	High-end	Δ (High–Mid)	Discordant pairs	p-value (exact)
Sigmoid segment analysis					
Sensitivity	83.2%	82.3%	−1.0% (−10.2 to +8.3)	b1 = 7, c1 = 6	1.00
Specificity	81.7%	86.5%	+4.8% (−4.5 to +14.1)	b2 = 9, c2 = 15	0.23
PPV	80.3%	84.5%	+4.2% (−4.6 to +13)	–	–
NPV	84.4%	84.5%	+0.1% (−8.9 to +9.1)	–	–
Accuracy	82.4%	84.1%	+1.7% (−5.2 to +8.6)	–	–

Metric (descending)	Mid-end	High-end	Δ (High–Mid)	Discordant pairs	p-value (exact)
Descending colon segment analysis					
Sensitivity	70.1%	74.6%	+4.5% (−7.6 to +16.7)	b1 = 2, c1 = 5	0.48
Specificity	84.2%	83.0%	−1.2% (−8.7 to +6.3)	b2 = 15, c2 = 13	0.84
PPV	63.5%	63.3%	−0.2% (−12.6 to +12.2)	–	–
NPV	87.8%	89.3%	+1.5% (−6.4 to +9.4)	–	–
Accuracy	80.3%	80.7%	+0.4% (−6.1 to +6.9)	–	–

Metric (transverse)	Mid-end	High-end	Δ (High–Mid)	Discordant pairs	p-value (exact)
Transverse colon segment analysis					
Sensitivity	75.6%	78.0%	+2.4% (−11.3 to +16.2)	b1 = 2, c1 = 3	1.00
Specificity	97.5%	96.4%	−1.1% (−3.5 to +1.5)	b2 = 3, c2 = 1	0.63
PPV	86.1%	82.1%	−4.0% (−18.6 to +10.5%)	–	–
NPV	95.2%	95.5%	+0.3% (−5.8 to +6.5)	–	–
Accuracy	93.7%	93.3%	−0.4% (−4.1 to +3.3)	–	–

Metric (ascending)	Mid-end	High-end	Δ (High–Mid)	Discordant pairs	p-value (exact)
Ascending colon segment analysis					
Sensitivity	65.4%	76.9%	+11.5% (−22.6 to +42.7)	b1 = 2, c1 = 5	0.48
Specificity	96.7%	96.2%	−0.5% (−5.7 to +4.8)	b2 = 3, c2 = 2	1.00
PPV	70.8%	71.4%	+0.6% (−32.1 to +33.9)	–	–
NPV	95.7%	97.1%	+1.4% (−3.9 to +6.6)	–	–
Accuracy	93.2%	94.0%	+0.8% (−5.5 to +7.2)	–	–

Metric (caecum)	Mid-end	High-end	Δ (High–Mid)	Discordant pairs	p-value (exact)
Cecum segment analysis					
Sensitivity	62.5%	70.8%	+8.3% (−18.3 to +34.9)	b1 = 2, c1 = 4	0.68
Specificity	96.7%	97.2%	+0.5% (−2.8 to +3.8)	b2 = 2, c2 = 3	1.00
PPV	68.2%	73.9%	+5.7% (−20.7 to +32.1)	–	–
NPV	95.8%	96.7%	+0.9% (−2.7 to +4.5)	–	–
Accuracy	93.2%	94.5%	+1.3% (−3 to +5.6)	–	–

Differences between platforms (Δ), and exact McNemar p-values are provided. Discordant pairs: b1 = mid-end positive/high-end negative; c1 = mid-end negative/high-end positive; b2 = mid-end negative/high-end positive; c2 = mid-end positive/high-end negative.

**Table 3:** Overall and segment-wise diagnostic performance comparing mid-end and high-end intestinal ultrasound systems in Crohn's disease across the whole colon and individual segments.

Metric/Segment	Mid-end	High-end	Delta	Discordant	p-value
Overall segment analysis					
Metric	Mid-end	High-end	Δ (High–Mid)	Discordant pairs	p-value
Sensitivity	85.1%	87.6%	+2.5% (−8.2 to 13)	5/10	0.2
Specificity	62.0%	60.0%	−2.0% (−28 to +24.2)	4/3	0.7
PPV	87.8%	87.6%	−0.2% (−10.5 to +10.1)	–	–
NPV	56.3%	60.0%	+3.7% (−22.4 to 29.1)	–	–
Accuracy	79.6%	81.0%	+1.4% (−9.3 to 12.1)	–	–

Metric	Mid-end	High-end	Δ (High–Mid)	Discordant pairs	p-value
Ileum segment analysis					
Sensitivity	83.5%	87.2%	+3.7% (−5.7 to +13.1)	1/4	0.18
Specificity	82.1%	81.0%	−1.1% (−12.9 to +10.5)	5/4	0.74
PPV	85.8%	85.6%	−0.2% (−9.6 to +9)	–	–
NPV	79.3%	82.9%	+3.6% (−8.2 to +15.4)	–	–
Accuracy	82.9%	84.4%	+1.5% (−5.8 to +8.9)	–	–

Metric	Mid-end	High-end	Δ (High–Mid)	Discordant pairs	p-value
Right colon segment analysis					
Sensitivity	81.5%	87.0%	+5.5% (−8.1 to +19.3)	0/3	0.083
Specificity	78.9%	81.6%	+2.7% (−6.3 to +11.6)	9/13	0.39
PPV	57.9%	62.7%	+4.8% (−10.7 to +20.3)	–	–
NPV	92.3%	94.7%	+2.4% (−3.6 to +8.4)	–	–
Accuracy	79.6%	83.0%	+3.4% (−4.2 to +10.9)	–	–

Metric	Mid-end	High-end	Δ (High–Mid)	Discordant pairs	p-value
Transverse colon segment analysis					
Sensitivity	74.2%	71.0%	−3.2% (−25.4 to +19)	1/0	0.32
Specificity	97.2%	94.4%	−2.8% (−7 to 1.4)	7/2	0.095
PPV	82.1%	68.8%	−13.3% (−34.8 to +8)	–	–
NPV	95.6%	94.9%	−0.7% (−7.7 to +6.3)	–	–
Accuracy	93.8%	90.9%	−2.9% (−10 to +4.2)	–	–

Metric	Mid-end	High-end	Δ (High–Mid)	Discordant pairs	p-value
Left colon segment analysis					
Sensitivity	76.6%	78.7%	+2.1% (−13.7 to +17.9)	0/0	–
Specificity	89.6%	93.3%	+3.7% (−1.3 to +8.7)	5/11	0.13
PPV	67.9%	77.1%	+9.2% (−9.5 to +27.9)	–	–
NPV	93.1%	93.9%	+0.8% (−5.4 to +7)	–	–
Accuracy	86.7%	90.1%	+3.4% (−1.3 to +8.1)	–	–

Metric	Mid-end	High-end	Δ (High–Mid)	Discordant pairs	p-value
Rectum segment analysis					
Sensitivity	84.6%	82.0%	−2.6% (−17.4 to +12.3)	1/0	0.32
Specificity	93.0%	92.4%	−0.6% (−3.9 to +2.8)	7/6	0.78
PPV	73.3%	71.1%	−2.2% (−18.8 to +14.4)	–	–
NPV	96.4%	95.8%	−0.6% (−3.5 to +2.3)	–	–
Accuracy	91.4%	90.5%	−0.9% (−6.4 to +4.5)	–	–

Metric	Mid-end	High-end	Δ (High–Mid)	Discordant pairs	p-value
Complications detected					
Sensitivity	75.0%	85.0%	+10.0% (−6.3 to +45.9)	0/2	0.16
Specificity	100.0%	100.0%	+0.0% (0–0)	0/0	–
PPV	100.0%	100.0%	+0.0% (0–0)	–	–
NPV	97.4%	98.5%	+1.0% (−3.9 to +6)	–	–
Accuracy	97.6%	98.6%	+0.9% (−2.4 to +4.2)	–	–

Differences between platforms (Δ), and exact McNemar p-values are provided. Discordant pairs: b1 = mid-end positive/high-end negative; c1 = mid-end negative/high-end positive; b2 = mid-end negative/high-end positive; c2 = mid-end positive/high-end negative. Complication detection performance is also shown.

### Discriminative performance

#### Ulcerative colitis

**Fig. 2:**
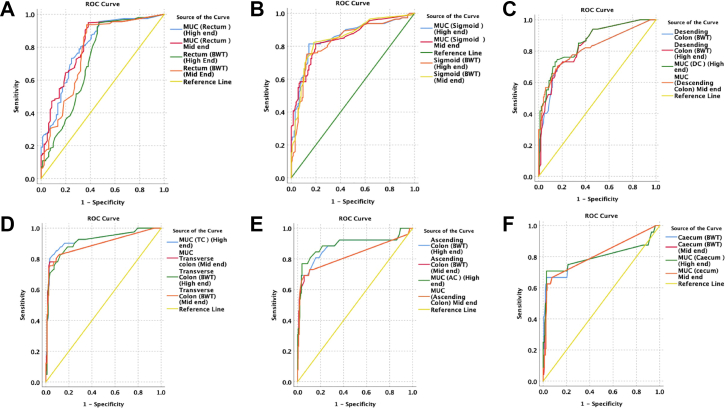
Segment-wise ROC curves for UC (High-end versus Mid-end IUS) ROC curves comparing bowel wall thickness (BWT) and Milan ultrasound criteria (MUC) obtained using high-end and mid-end IUS across six colonic segments—rectum (A), sigmoid colon (B), descending colon (C), transverse colon (D), ascending colon (E), and caecum (F). Both systems show similar discriminative performance for detecting endoscopic activity, with AUCs consistently in the good–excellent range. Curves demonstrate minimal separation between platforms and markers.

In ulcerative colitis, discriminative performance of mid-end and high-end systems was similar across all colonic segments (Supplementary Table S5; Fig. 2). For rectal disease, the AUC for BWT was 0.77 (95% CI 0.70–0.84) with the mid-end system and 0.72 (95% CI 0.65–0.80) with the high-end system, while rectal MUC showed AUCs of 0.81 (95% CI 0.75–0.87) and 0.80 (95% CI 0.74–0.86), respectively. In the sigmoid colon, BWT AUCs were 0.85 (95% CI 0.80–0.90) for mid-end and 0.83 (95% CI 0.78–0.89) for high-end ultrasound, with corresponding MUC AUCs of 0.85 (95% CI 0.80–0.90) and 0.85 (95% CI 0.80–0.90).

Across the descending, transverse, ascending colon, and caecum, BWT and MUC demonstrated consistently good discrimination with overlapping confidence intervals between systems. Overall MUC performance showed an AUC of 0.85 (95% CI 0.80–0.90) for the mid-end system and 0.83 (95% CI 0.77–0.89) for the high-end system. Excluding the rectum, overall, MUC discrimination remained similar (mid-end AUC 0.77 [95% CI 0.71–0.83] versus high-end AUC 0.83 [95% CI 0.77–0.89]) (Supplementary Figure S2). No colonic segment demonstrated a statistically significant difference in AUC between platforms.

#### Crohn's disease

**Fig. 3:**
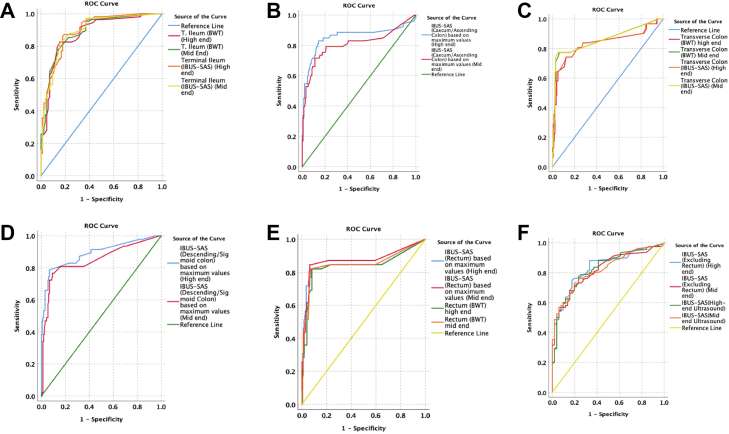
Segment-wise ROC curves for Crohn's disease (High-end versus Mid-end IUS). ROC curves comparing bowel wall thickness (BWT) and IBUS-SAS across major CD-affected segments—terminal ileum (A), right colon (caecum/ascending) (B), transverse colon (C), descending/sigmoid colon (D), rectum (E), and overall/rectum-excluded IBUS-SAS (F). Curves show consistently high AUCs with minimal separation between mid-end and high-end systems, indicating comparable discriminative accuracy across all bowel regions for detecting endoscopic activity.

In Crohn's disease, both systems demonstrated comparable discriminative performance across ileocolonic segments for BWT and IBUS-SAS (Supplementary Table S6; Fig. 3). In the terminal ileum, BWT yielded AUCs of 0.89 (95% CI 0.84–0.94) with the mid-end system and 0.88 (95% CI 0.83–0.93) with the high-end system, while IBUS-SAS showed AUCs of 0.89 (95% CI 0.85–0.94) and 0.90 (95% CI 0.85–0.94), respectively.

For the caecum/ascending colon, IBUS-SAS based on maximum segmental values demonstrated AUCs of 0.81 (95% CI 0.73–0.89) for mid-end and 0.86 (95% CI 0.78–0.93) for high-end ultrasound. In the transverse colon, BWT AUCs were 0.86 (95% CI 0.78–0.94) for mid-end and 0.83 (95% CI 0.73–0.93) for high-end systems, with similar findings for IBUS-SAS.

Rectal discrimination was also comparable, with BWT AUCs of 0.85 (95% CI 0.76–0.94) for mid-end and 0.84 (95% CI 0.75–0.93) for high-end ultrasound, and IBUS-SAS AUCs of 0.87 (95% CI 0.79–0.96) and 0.85 (95% CI 0.76–0.95), respectively. Overall IBUS-SAS discrimination was nearly identical between platforms (mid-end AUC 0.82 [95% CI 0.76–0.88] versus high-end AUC 0.83 [95% CI 0.76–0.89]), including analyses excluding the rectum.

#### Impact on management

In UC, management changed in 19/239 patients (7.9%): 13 were correct (5 true-positive escalations, 8 true-negative de-escalations) and 9 were incorrect (8 false-positive, 1 false-negative), yielding an NRI of +4 patients (1.7%) and a management DOR of 5.0 (95% CI: 0.47–53), without statistical significance (p = 0.08). In CD, management changed in 24/211 patients (11.4%): 13 correct (10 true-positive, 3 true-negative) and 11 incorrect (5 false-positive, 6 false-negative), producing an NRI of +2 patients (0.95%) and a DOR of 1.0 (95% CI: 0.18–5.6), indicating no meaningful advantage over the mid-end system.

Detailed case-level review showed that in UC, 7 of the 8 false-positive cases were attributable to exaggerated Doppler vascular signal detection on the high-end system without corresponding endoscopic inflammation. One false-positive and the single false-negative case were related to rectal assessment variability.

In CD, 5 false-positive cases were similarly driven by overestimation of vascular activity. Among the 6 false-negative cases, 2 were due to very mild endoscopic disease without significant transmural thickening, 1 was related to rectal assessment limitations, and in 3 cases subtle inflammatory changes were not detected sonographically despite adequate image acquisition. No consistent segmental or technical failure pattern was observed.

### Agreement and concordance between systems

#### Ulcerative colitis

**Fig. 4:**
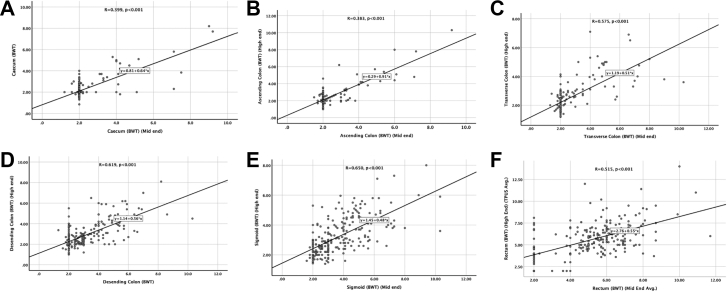
Segment-wise correlation between mid-end and high-end intestinal ultrasound bowel wall thickness (BWT) measurements in ulcerative colitis. Scatter plots with linear regression for BWT in the caecum (A), ascending colon (B), transverse colon (C), descending colon (D), sigmoid colon (E), and rectum (F), demonstrating positive correlation between systems with modest dispersion across segments.

In UC, segment-wise agreement between mid-end and high-end systems for bowel wall thickness (BWT) measurements showed moderate-to-high concordance across all colonic segments (Supplementary Table S7; Fig. 4). Lin's concordance correlation coefficient (CCC) for BWT was 0.54 in the rectum (precision r = 0.55; C_β_ = 0.98), 0.59 in the sigmoid colon (r = 0.64; C_β_ = 0.91), 0.66 in the descending colon (r = 0.68; C_β_ = 0.97), and 0.65 in the transverse colon (r = 0.68; C_β_ = 0.96). Higher concordance was observed in the ascending colon (CCC = 0.83; r = 0.83; C_β_ = 0.99) and caecum (CCC = 0.76; r = 0.77; C_β_ = 0.98).

Bland–Altman analyses demonstrated small mean differences between systems with narrow limits of agreement across segments, indicating minimal fixed bias (Supplementary Figure S3). Segment-wise concordance for Milan Ultrasound Criteria (MUC) followed similar patterns (Supplementary Figures S4–S7).

#### Crohn's disease

In CD, agreement between mid-end and high-end systems for segmental measurements was similarly consistent across ileocolonic segments (Supplementary Table S8; Supplementary Figures S8 and S9). For bowel wall thickness and IBUS-SAS–based assessments, CCC values were 0.54 in the terminal ileum (r = 0.69; C_β_ = 0.77), 0.61 in the caecum (r = 0.72; C_β_ = 0.84), and 0.66 in the ascending colon (r = 0.69; C_β_ = 0.96). Concordance was higher in the transverse colon (CCC = 0.74; r = 0.78; C_β_ = 0.94) and rectum (CCC = 0.77; r = 0.77; C_β_ ≈ 1.00), with intermediate values observed in the descending (CCC = 0.58) and sigmoid colon (CCC = 0.59).

Bland–Altman plots showed small mean differences and no systematic over- or under-estimation between systems, although proportional bias was observed in selected segments (Supplementary Figures S7 and S8). Segment-wise concordance for IBUS-SAS scores followed similar patterns (Supplementary Figures S10 and S11).

#### Clinical interchangeability of bowel wall thickness measurements

Clinical acceptability of bowel wall thickness (BWT) measurements, defined a priori as an inter-system difference within ±1 mm, was high across most ileocolonic segments in both UC and CD (Supplementary Tables S9 and S10). In UC, the proportion of clinically acceptable paired measurements was 92.5% in the caecum and ascending colon, 90.0% in the transverse colon, 79.5% in the descending colon, and 69.5% in the sigmoid colon. Acceptability was lowest in the rectum (54.2%), where wider limits of agreement were observed. Mean inter-system differences were small across all segments, ranging from −0.005 mm in the caecum to −0.62 mm in the rectum, with segment-specific limits of agreement reflecting greater variability in distal colonic measurements.

In CD, clinical acceptability exceeded 87% across all segments. Acceptable agreement was observed in 87.2% of terminal ileal measurements, 93.8% in the caecum, 95.2% in the ascending colon, 95.7% in the transverse and descending colon, 97.2% in the sigmoid colon, and 90.5% in the rectum. Mean inter-system differences were small across segments, and corresponding Lin's concordance correlation coefficients indicated moderate agreement, consistent with the observed limits of agreement (Supplementary Tables S9 and S10). Even segments with greater variability, such as the rectum, maintained acceptable agreement, supporting the clinical interchangeability of both platforms across the entire ileocolon.

#### Decision curve analysis (DCA)

In UC, DCA showed similar net benefit for mid-end and high-end MUC-based models across a wide range of threshold probabilities (Supplementary Table S11; Supplementary Figure S12A). At lower thresholds (0.05–0.30), net benefit values were identical or nearly identical between systems (e.g., 0.71 versus 0.71 at threshold 0.05; 0.62 versus 0.62 at threshold 0.30). At intermediate thresholds (0.35–0.70), small numerical differences were observed, with net benefit ranging from 0.60 to 0.37 for the mid-end model and 0.62–0.42 for the high-end model. At higher thresholds (≥0.75), net benefit declined for both platforms, with overlapping or closely spaced values throughout. Across all thresholds, both ultrasound-based models yielded higher net benefit than the “treat-none” strategy and comparable or higher net benefit than the “treat-all” strategy.

In CD, net benefit values for mid-end and high-end IUS models were also closely aligned across threshold probabilities (Supplementary Table S12; Supplementary Figure S12B). From thresholds 0.05 to 0.50, net benefit differed minimally between platforms (e.g., 0.64 versus 0.64 at threshold 0.05; 0.55 versus 0.54 at threshold 0.50). At intermediate thresholds (0.55–0.75), net benefit gradually declined for both systems, with small absolute differences. At higher thresholds (≥0.80), net benefit became negative for both models. Across the full threshold range, both models consistently outperformed the “treat-none” strategy and showed similar performance relative to the “treat-all” strategy.

## Discussion

In this large, prospective, paired study of 450 patients with UC and CD, a mid-end IUS system performed similar to a high-end system across all key diagnostic and clinical domains. Sensitivity for detecting active inflammation was high in both UC (>94%) and CD (85–88%) with no significant absolute differences, and specificity was similarly modest yet comparable between platforms. ROC analyses showed near-superimposable AUCs for bowel wall thickness and validated activity scores (MUC in UC; IBUS-SAS in CD) across all ileocolonic segments. Quantitative measurements demonstrated high concordance, with correlation coefficients typically 0.50–0.80, substantial CCC values, and >90% of inter-system BWT differences falling within ±1 mm in most segments. Complication detection in CD was excellent for both platforms, and decision-curve analysis revealed overlapping net benefit, indicating no additional decision-making advantage of the high-end device. Although the high-end system produced numerically more correct reclassifications, net reclassification improvements were very small (UC +1.7%; CD +0.9%) and not clinically meaningful. Collectively, these findings show that mid-end IUS is clinically interchangeable with high-end systems for routine assessment and monitoring in IBD.

Several studies including systematic reviews have consistently shown strong correlation between IUS parameters and endoscopic severity, with sensitivity of 80–95% and specificity of 60–85% across segments.^1^ These findings likely reflect the known limitation of IUS in detecting superficial mucosal lesions without associated transmural inflammatory changes. Findings from the HUMID study not only confirm these ranges, but also extend them by demonstrating hardware-equivalence, an evidence gap not previously addressed. A large, prospective study has suggested that modern mid-range systems could reliably predict endoscopic activity, but no prospective, paired, same-patient comparisons were conducted earlier.^8^ In CD, diagnostic accuracy in the terminal ileum was numerically higher than the overall segmental performance, whereas some colonic segments demonstrated lower accuracy; however, these differences were not statistically significant. The predominance of mild lesions, mixed phenotypic distribution, incorporation of transperineal ultrasound for rectal assessment, and use of contemporary high-resolution systems may have attenuated the ileum–colon gradient described in earlier cohorts.^1^ Segment-level precision was also limited by sample size.

Multiple studies have established IUS as a reliable tool for assessing luminal inflammation in both UC and CD. The Milan Ultrasound Criteria (MUC) for UC, validated across several cohorts, consistently show strong correlation between BWT, hyperaemia, and endoscopic activity, with reported AUCs of 0.82–0.94. Our UC results (AUCs 0.75–0.93) closely align with these benchmarks and showed no meaningful difference between systems.^18^ Likewise, the IBUS-SAS—developed through Delphi consensus and multicentre reliability testing—demonstrates excellent reproducibility, with near-perfect agreement for BWT (ICC 0.96).^19^ In our HUMID study, mid-end and high-end systems produced virtually identical IBUS-SAS values and ROC curves, confirming that this validated CD activity score is not impacted by hardware platform differences. Case-level review demonstrated that most false positives were driven by exaggerated vascular signal detection, whereas false negatives were largely confined to mild mucosal disease or rectal assessment limitations.

IUS is now central to treat-to-target strategies, as endorsed by STRIDE-II.^20^ Prior prospective studies—including TRUST and work by Maaser et al.—have shown that changes in BWT and doppler reliably track treatment response and mucosal healing.^21^ Prospective cohort studies further demonstrate that IUS-guided early management improves outcomes and reduces reliance on endoscopy.^8^^,^^22^ The HUMID study extends this evidence by showing that hardware choice does not influence management accuracy: the very small NRIs (+1–2%) were clinically negligible, confirming that mid-end systems are completely adequate for escalation, de-escalation, and ongoing monitoring decisions.

Although formal cost-effectiveness modelling was beyond the scope of this study, previously reported market data indicate that refurbished mid-end systems (e.g., Siemens ACUSON S2000) are available at approximately 1575–13,781 USD, whereas new high-end platforms with dedicated intestinal ultrasound capability range from 62,181 to 65,598 USD.^8^ Given the comparable diagnostic performance observed in HUMID, these findings suggest a capital cost differential exceeding 50,000 USD per unit, potentially lowering barriers to implementation in resource-variable health systems.

This study has several strengths. It represents one of the largest prospective, paired IUS datasets, with 450 patients undergoing same-patient, same-day scans on both platforms—eliminating temporal variability and enabling a true head-to-head comparison. Inclusion of both UC and CD permitted disease- and segment-specific evaluation across the entire ileocolon. Blinded cross-platform scanning minimised interpretation bias, and the comprehensive analytic framework—diagnostic accuracy, quantitative agreement, complication detection, ROC performance, and decision-curve analysis—provides a multidimensional assessment of hardware equivalence in IUS. The consistently narrow limits of agreement for BWT, strong CCC values, and overlapping ROC and DCA curves collectively provide strong evidence that high diagnostic accuracy in IUS does not require a high-end system, provided acquisition protocols and operator expertise are standardised-a principle also supported by earlier work demonstrating comparable performance even with handheld ultrasound devices.^11^ Importantly, the cohort reflected real-world clinical phenotypes rather than trial-selected populations, enhancing the generalisability of our findings.

This study has a few limitations. As a single-centre evaluation performed by experienced IUS operators, results may overestimate performance in less-experienced hands, though the high agreement between systems suggests reproducibility is largely biological rather than centre-specific. In addition, each operator performed examinations exclusively on their routinely assigned ultrasound platform to reflect institutional workflow. While this approach mirrors real-world practice, a potential operator–platform effect cannot be completely excluded despite protocol standardisation and high agreement observed during the pilot benchmarking phase. Specificity—particularly in UC—was modest, reflecting intrinsic challenges of distinguishing chronic change from active inflammation rather than hardware differences. Only about 55% of participants underwent ileocolonoscopy based on clinical indication and IUS findings, which may limit generalisability to surveillance settings. Because colonoscopy was guided by the high-end IUS examination within routine care pathways, a theoretical risk of verification bias cannot be entirely excluded, and the analysed cohort may represent a clinically enriched population with a higher probability of active disease. Nevertheless, all analysed patients underwent complete paired verification against a blinded reference standard, supporting the internal validity of the platform comparison while limiting estimation of population-level diagnostic accuracy. The classification of systems as “mid-end” and “high-end” reflects pragmatic market segmentation rather than formal manufacturer-defined categories, which may limit universal applicability of this terminology. Because scan order was not randomised (mid-end followed by high-end), an order effect cannot be fully excluded, although independent operators and blinding minimised the risk of systematic reporting bias. SES-CD component subscores (including ulcer subscores) in CD were not recorded in a format that allowed reconstruction of an ulcer-free endpoint; therefore, we could not perform a sensitivity analysis using ulcer-free remission definitions. Future benchmarking studies should prospectively capture SES-CD component subscores to enable ulcer-focused endpoints. Although formal ICCs were not calculated for the main comparative analysis, multiple complementary agreement methods (correlation, Bland–Altman, CCC, and clinically anchored acceptability thresholds) provide a rigorous and multidimensional assessment appropriate for hardware-comparison studies targeting interchangeability rather than inter-observer reproducibility. Independent expert panels for mid-end IUS, high-end IUS, and reference endoscopic assessments may introduce inter-panel variability in decision thresholds. While separate panels were intentionally used to minimise recall and anchoring bias, panel-related variability represents another potential source of unmeasured confounding in management decision analyses. This design was chosen to minimise recall and anchoring bias and to reflect real-world multidisciplinary decision-making; correctness of reclassification was anchored to an independent ileocolonoscopy-based reference standard. Finally, while cost considerations motivated the research, formal economic modelling was beyond the scope of this study; nonetheless, the observed hardware equivalence offers a strong rationale for future cost-effectiveness analyses. While intestinal ultrasound is well validated for proximal small-bowel CD, this phenotype was intentionally excluded from HUMID to avoid reference-standard heterogeneity; these disease locations have been examined in prior work from our group.^12^ Taken together, these methodological considerations highlight that the HUMID study primarily provides a pragmatic head-to-head comparison of ultrasound platforms within a real-world clinical workflow. Therefore, the findings should be interpreted in the context of these study conditions and potential sources of bias.

These findings have important clinical and system-level implications. Our results suggest that mid-end ultrasound systems may be clinically interchangeable with high-end platforms within the study conditions, potentially removing a major barrier to IUS adoption, particularly in resource-limited settings. Treat-to-target strategies may therefore be feasible using widely available mid-range devices without compromising diagnostic accuracy or therapeutic decision-making, shifting the emphasis from hardware sophistication to operator training, standardised acquisition, and streamlined workflow integration. At the health-system level, these results support the potential for scalable, cost-efficient deployment of IUS and align with ECCO and IBUS initiatives to expand global access to point-of-care imaging. However, these findings should be interpreted in the context of the study design and require confirmation in broader practice settings.

Future studies should focus on multicentre validation, evaluation across varying operator experience, cost-effectiveness analyses, and emerging standardisation tools, including automated and artificial intelligence–assisted IUS.

## Contributors

Partha Pal (Conceptualisation: Lead; Investigation: Equal; Methodology: Lead; Data curation-equal, Formal Analysis: Lead; Supervision: Lead; Validation: Lead; Visualisation: Lead; Writing—original draft, Review & editing: Lead).

Mohammad Abdul Mateen (Investigation: Equal; Project administration: supporting; Resources: Supporting; Supervision: Supporting; Methodology: equal; Writing—review & editing: equal).

Syed Misba Naaz (Data curation: Equal; Formal analysis: supporting; Methodology: equal; Writing—original draft: supporting; Writing—review & editing: supporting).

Kanapuram Pooja (Data curation: Equal; Formal analysis: supporting; Methodology: equal; Writing—original draft: supporting; Writing—review & editing: supporting).

Radhika Nittala (Investigation: supporting; Writing—review & editing: supporting).

Ayush Singh (Investigation: supporting; Writing—review & editing: supporting).

Bharath Kumar Reddy (Investigation: supporting; Writing—review & editing: supporting).

Praveen Kumar Reddy Vasepally (Investigation: supporting; Writing—review & editing: supporting).

Sayyed Mahiboob Najbuddin (Investigation: supporting; Writing—review & editing: supporting).

Tummalapalli Satya Maharshi (Investigation: supporting; Writing—review & editing: supporting).

Uday Kumar Marri (Investigation: supporting; Writing—review & editing: supporting).

Zaheer Nabi (Investigation: supporting; Resources: equal, Writing—review & editing: supporting).

Palle Manohar Reddy (Investigation: supporting; Writing—review & editing: supporting).

Jahangeer Basha (Investigation: supporting; Writing—review & editing: supporting).

Rajesh Gupta (Project administration: equal; Investigation: supporting; Writing—review & editing: supporting).

Manu Tandan (Project administration: equal; Resources: equal, Investigation: supporting; Writing—review & editing: supporting).

Rupjyoti Talukdar (conceptualisation: equal; Writing-review and editing: equal; Statistical review: supporting).

D Nageshwar Reddy (Project administration: Lead; Resources: Lead, Investigation: supporting; Writing—review & editing: supporting).

Partha Pal, Syed Misba Naaz, and Kanapuram Pooja accessed and verified the underlying data.

## Data sharing statement

The datasets generated during and/or analysed during the current study are available from the corresponding author on reasonable request.

## Declaration of interests

Partha Pal received consultancy fees/speaker honorarium from Johnson and Johnson, Takeda Pharmaceutical Company, Cipla Ltd, Sun Pharma, Zydus Biosciences and Dr Reddy Labs. Other authors disclose no conflicts.

## Appendix A Supplementary data

Supplementary Material

Protocol
