# Supplementary material for: High-end intestinal ultrasound versus mid-end systems benchmarked against tandem ileocolonoscopy in inflammatory bowel disease (HUMID): a paired prospective, validating confirmatory study
Source: eClinicalMedicine. 2026 Apr 1;94:103856. doi: 10.1016/j.eclinm.2026.103856 (PMC13068867; doi:10.1016/j.eclinm.2026.103856)
Supplement: Supplementary Material [file mmc1.docx]

**Supplementary material**

**Combined intestinal ultrasound protocol (HUMID study)**

All participants with established inflammatory bowel disease underwent a standardized clinical and ultrasound assessment as part of routine care at the inflammatory bowel disease clinic. Clinical activity was assessed using the Simple Clinical Colitis Activity Index for ulcerative colitis and the Harvey–Bradshaw Index for Crohn’s disease by trained clinical research associates.

**Mid-end and high-end transabdominal intestinal ultrasound**

Transabdominal intestinal ultrasound was performed without bowel preparation, with patients examined in the supine position. Each participant first underwent examination on a mid-end ultrasound system, followed immediately by a high-end ultrasound examination in the same session, using a fixed scan order. Operators were blinded to the findings of the alternate system.

The mid-end intestinal ultrasound examinations in the HUMID study were performed using a pre-existing mid-range ultrasound platform (Siemens ACUSON S2000, Siemens Healthcare, Erlangen, Germany), following a standardized acquisition and reporting protocol adapted from our previously published prospective real-world experience.[1]All scans were conducted using a high-frequency linear transducer (9L4; 4–9 MHz), with effective bowel imaging achieved at frequencies ≥7 MHz. In the absence of a dedicated bowel ultrasound preset, optimized paediatric abdominal (7 MHz) or thyroid (8 MHz) presets with a trapezoid field of view were used to enhance bowel loop visualization, spatial resolution, and delineation of transmural and mesenteric features.[1]

High-end examinations were performed using advanced bowel presets in Samsung RS80 EVO (Samsung India Electronics Pvt. Ltd, Haryana, India) with low-frequency convex probes (approximately 1–6 MHz) for global assessment, followed by high-frequency linear probes (2–9 MHz and 2-14 MHz) for detailed mural evaluation. A standardized scanning sequence was followed in all cases, beginning in the sigmoid colon and progressing proximally through the descending, transverse, and ascending colon to the cecum, with subsequent assessment of the terminal ileum. Each segment was examined in longitudinal and transverse planes. Bowel wall thickness was measured at the site of maximal thickening, avoiding folds and luminal compression, using the mean of repeated orthogonal measurements.

High-end ultrasound platforms are distinguished from mid-range systems by multiple hardware-level enhancements. These include markedly greater computational performance (often expressed in billion operations per second), enabling advanced real-time rendering, motion analysis, and quantitative processing. High-end systems frequently incorporate single-crystal transducer technology, which improves signal transmission efficiency and uniformity compared to conventional piezoelectric ceramic probes used in mid-range devices. They offer higher spatial resolution, improved delineation of fine mural architecture, superior contrast optimization through advanced speckle suppression and edge enhancement algorithms, and more consistent image quality across depth. Broader frequency bandwidth options (e.g., 2–14 MHz versus 4–12 MHz) allow better flexibility between penetration and resolution. Dedicated bowel presets and enhanced Doppler sensitivity may facilitate shorter acquisition times and easier workflow integration. These technical refinements are associated with substantially higher procurement costs, whereas mid-range systems typically utilize existing installed equipment without additional capital investment (supplemtary table 1).[1]

Inflammatory activity was assessed using predefined sonographic criteria, including bowel wall thickness, mural stratification, colour Doppler vascularity, inflammatory mesenteric fat, lymphadenopathy, and complications. Colour Doppler vascularity was graded using the modified Limberg score with low-flow settings (5–8 cm/s), adjusting gain to the threshold of background noise. Disease activity was quantified using the Milan Ultrasound Criteria for ulcerative colitis and the International Bowel Ultrasound Segmental Activity Score for Crohn’s disease.

**Optimisation of the Mid-End Ultrasound System**

To ensure optimal bowel visualisation and fair comparison with the high-end system, several predefined technical optimisations were applied to the mid-end ultrasound platform, as previously described in our prospective real-world study using the same equipment.[1]

**Probe selection and frequency:**
All examinations were performed using a high-frequency linear transducer (9L4; 4–9 MHz), with imaging conducted at frequencies ≥7 MHz whenever feasible to maximise spatial resolution for bowel wall assessment.

**Preset adaptation:**
In the absence of a dedicated bowel preset, paediatric abdominal (7 MHz) and thyroid (8 MHz) presets were selectively employed. These presets were chosen to enhance near-field resolution, improve contrast differentiation of bowel wall layers, and facilitate delineation of surrounding inflammatory fat, as demonstrated in prior work.

**Field-of-view and image geometry:**
A trapezoid (expanded) field-of-view was routinely enabled to allow complete visualisation of bowel loops in both longitudinal and transverse planes, particularly for terminal ileal and colonic segments.

**Gain, depth, and focus optimisation:**
Overall gain was reduced to avoid blooming artefact, with focal zones positioned at the level of the bowel wall. Imaging depth was minimised to optimise axial resolution, particularly in lean and average body habitus.

**Doppler optimisation:**
Colour Doppler settings were standardised across examinations, using low-flow settings (4–6 cm/s) with incremental gain adjustment until background noise suppression, in line with modified Limberg scoring recommendations.

**Documentation protocol:**
All pathological segments were documented using paired longitudinal and cross-sectional still images, supplemented by cine loops to capture peristalsis, vascularity, and segmental extent of disease.

These optimisations allowed reliable intestinal ultrasound assessment using a mid-end platform and formed the technical foundation for the comparative evaluation performed in the HUMID study.

**Transperineal ultrasound (rectal assessment)**

Transperineal ultrasound (TPUS) was additionally performed for detailed rectal assessment during the same session. Transabdominal visulaization of rectum was done in supine position with full urinary bladder using a low-frequency curvilinear ultrasound probe or linear probe in both longitudinal and axial section for both systems. Transperineal ultrasound was conducted after transabdominal scanning, using the same ultrasound platform (mid-end or high-end, as applicable).Transperineal ultrasound was performed with the patient in the left lateral position. A linear probe (4-9 MHz for mid end system and 2–9 MHz from high end system) was positioned anterior to the anus and covered with a protective sheath filled with ultrasound gel. The probe was covered with a wrap filled with transducer gel which was disinfected initially with dry tissue followed by compatible disinfectant (Avagard, 1.0% chlorhexidine gluconate + 61% ethanol, 3M, India) after each TPUS. Rectal wall thickness was calculated using an average of anterior and posterior wall thickness. Wall thickness was measured from mucosa-air interface to outer muscle layer of rectum. If anterior wall was not well visualized in TPUS due to air artefacts, then only posterior wall thickness was measured and considered. In addition to measuring total rectal wall thickness. Vascularization was graded in TPUS using modified Limberg scale using flow 5–8 m/s and adjusting gain until the fading of noise.[2]

**Standardised definitions and reporting framework (HUMID study)**

All intestinal ultrasound examinations in the HUMID study were acquired, interpreted, and reported using predefined, consensus-based definitions to ensure reproducibility, cross-platform comparability, and suitability for use in clinical trials.

**Bowel wall thickness**

Bowel wall thickness was defined as the distance from the luminal mucosa–interface to the outer border of the muscularis propria. Measurements were performed in a relaxed, non-contracted bowel loop, avoiding haustral folds, peristaltic contractions, or external compression by the probe. Thickness was measured in both longitudinal and transverse planes, and the reported value represented the mean of at least two orthogonal measurements at the site of maximal thickening for each segment. For the colon and terminal ileum, a bowel wall thickness >3 mm was considered abnormal; for rectal assessment using transperineal ultrasound, a threshold >4 mm was used to account for physiological rectal wall thickness.[3, 4]

**Bowel wall stratification**

Mural stratification was assessed visually on high-frequency linear transducers and graded using the International Bowel Ultrasound Segmental Activity Score (IBUS-SAS) on a four-point ordinal scale reflecting the degree and longitudinal extent of disruption: Score 0 (Normal): Normal, multilayered bowel wall appearance with all layers clearly visible and well delineated. Score 1 (Uncertain): Indeterminate or blurred layer borders, with the third (submucosal) layer still identifiable but not clearly demarcated. Score 2 (Focal loss): Localised disruption or loss of normal stratification involving a segment measuring ≤3 cm in longitudinal extent. Score 3 (Extensive loss): Extensive disruption or complete loss of stratification involving a segment >3 cm in longitudinal extent.[3, 5]

**Colour Doppler vascularity**

Mural vascularity was assessed using colour Doppler imaging with low-flow settings (velocity scale 5–8 cm/s) and gain adjusted to the point of background noise suppression. Vascularity was graded using the modified Limberg score: grade 0, no detectable mural flow; grade 1, single short Doppler signal; grade 2, multiple short signals within the bowel wall; grade 3, marked mural flow with long or branching signals. Grades ≥1 were considered abnormal and indicative of active inflammation.[5]

**Inflammatory mesenteric fat**

Inflammatory mesenteric fat was evaluated adjacent to the affected bowel segment and graded as follows: Score 0: No inflammatory fat; normal, compressible mesenteric fat with normal echogenicity. Score 1 (Uncertain): Mild or equivocal increase in echogenicity without clear expansion or loss of compressibility. Score 2: Definite inflammatory fat characterised by hyperechoic, non-compressible mesenteric fat surrounding the bowel loop, often associated with increased vascularity.

**Lymphadenopathy**

Mesenteric lymph nodes were considered pathological if they were oval or round, hypoechoic, measured ≥5 mm in short-axis diameter, and located adjacent to an inflamed bowel segment. Lymphadenopathy was recorded as present or absent for each segment.

**Complications**

Strictures were defined as a persistent bowel wall thickening (> 3 mm) associated with luminal narrowing (< 1cm or >50% reduction in luminal diameter at the narrowest point compared with an adjacent normal bowel loop) and proximal dilatation (>25 mm for small bowel or a clear increase in luminal diameter relative to an adjacent normal bowel loop), with or without prestenotic hyperperistalsis.[6] Fistulas were defined as hypoechoic, tubular tracts connecting two bowel loops or a bowel loop to another structure, often with Doppler signal. Abscesses were defined as hypoechoic or anechoic collections with irregular margins and posterior acoustic enhancement, with or without internal echoes.

**Segmental disease extent**

For ulcerative colitis, ultrasound assessment was performed segmentally (rectum, sigmoid, descending colon, transverse colon, ascending colon, cecum), and disease extent was classified based on the most proximal segment demonstrating sonographic activity. Ileal findings were recorded but excluded from correlation analyses because endoscopic ulcerative colitis indices do not assess ileal activity.
For Crohn’s disease, segmental assessment included terminal ileum, cecum/ascending colon (right colon), transverse colon, descending/sigmoid colon (left colon), and rectum. When multiple ultrasound segments mapped to a single endoscopic region, the segment with the highest activity score was used for analysis.

**Composite activity indices**

In ulcerative colitis, sonographic activity was quantified using the Milan Ultrasound Criteria, incorporating bowel wall thickness and colour Doppler vascularity.
In Crohn’s disease, activity was quantified using the International Bowel Ultrasound Segmental Activity Score, incorporating bowel wall thickness, mural stratification, colour Doppler signal, and inflammatory mesenteric fat. Scores were calculated per segment and as overall and rectum-excluded summary measures.

**Transperineal ultrasound (rectal assessment)**

For transperineal ultrasound, rectal wall thickness was measured from the mucosa–air interface to the outer border of the muscularis propria in the midline plane. Measurements were obtained from both anterior and posterior walls and averaged. When anterior wall visualisation was limited by intraluminal air, posterior wall thickness alone was used. Doppler vascularity was assessed using the same low-flow modified Limberg criteria as transabdominal scanning.

**Reporting standards**

All ultrasound findings were recorded in a structured reporting template capturing segment-wise measurements, categorical inflammatory features, composite activity scores, and presence of complications. Reporting was performed independently for mid-end and high-end systems using identical definitions and thresholds to ensure true system-level comparison.

**Supplementary figures**

**Supplementary** **Figure 1.** Representative case illustrating paired intestinal ultrasound assessment and reference ileocolonoscopy in the HUMID study. (A) Mid-end intestinal ultrasound demonstrating bowel wall thickening of the terminal ileum and caecum with increased vascularity (modified Limberg grade 2). (B) Corresponding high-end intestinal ultrasound showing similar bowel wall thickening and vascularity in the same segments.(C) Ileocolonoscopy revealing ulceration at the ileocaecal valve, confirming endoscopically active disease.
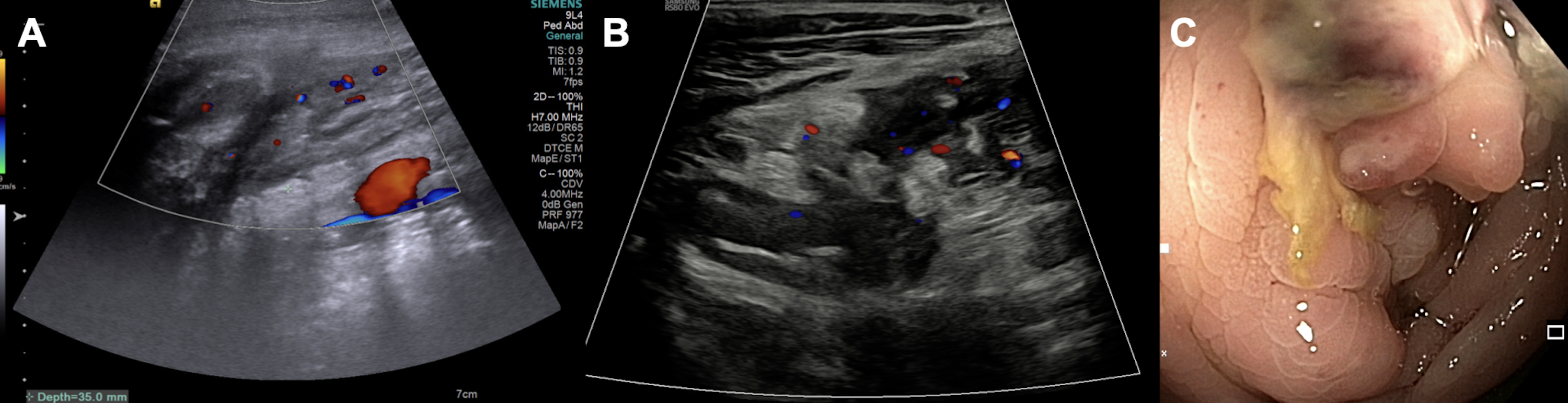


**Supplementary Figure 2**. Overall ROC curves for UC (High-end vs Mid-end IUS) Aggregate ROC analysis demonstrating overall and “excluding rectum” MUC scores for high-end and mid-end systems. Curves are nearly superimposable, indicating comparable global performance of both platforms for identifying active disease.
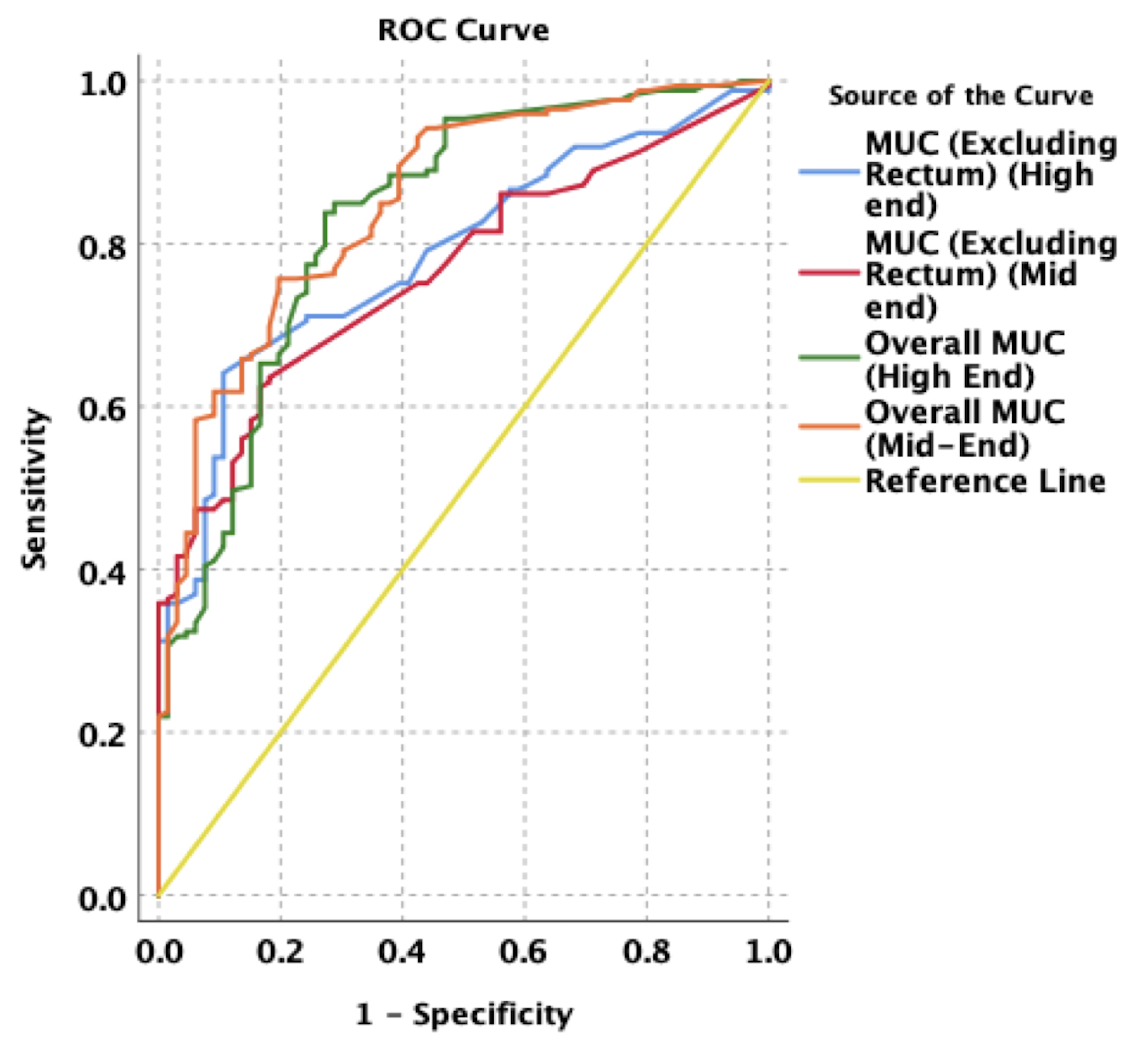


**Supplemntary Figure 3.** Bland–Altman agreement plots for bowel wall thickness (BWT) measured using mid-end and high-end ultrasound in ulcerative colitis. Plots show mean difference and 95% limits of agreement for the rectum (A), sigmoid (B), descending (C), transverse (D), ascending (E), and caecum (F). Differences were small and without segment-specific bias, indicating acceptable agreement between systems.
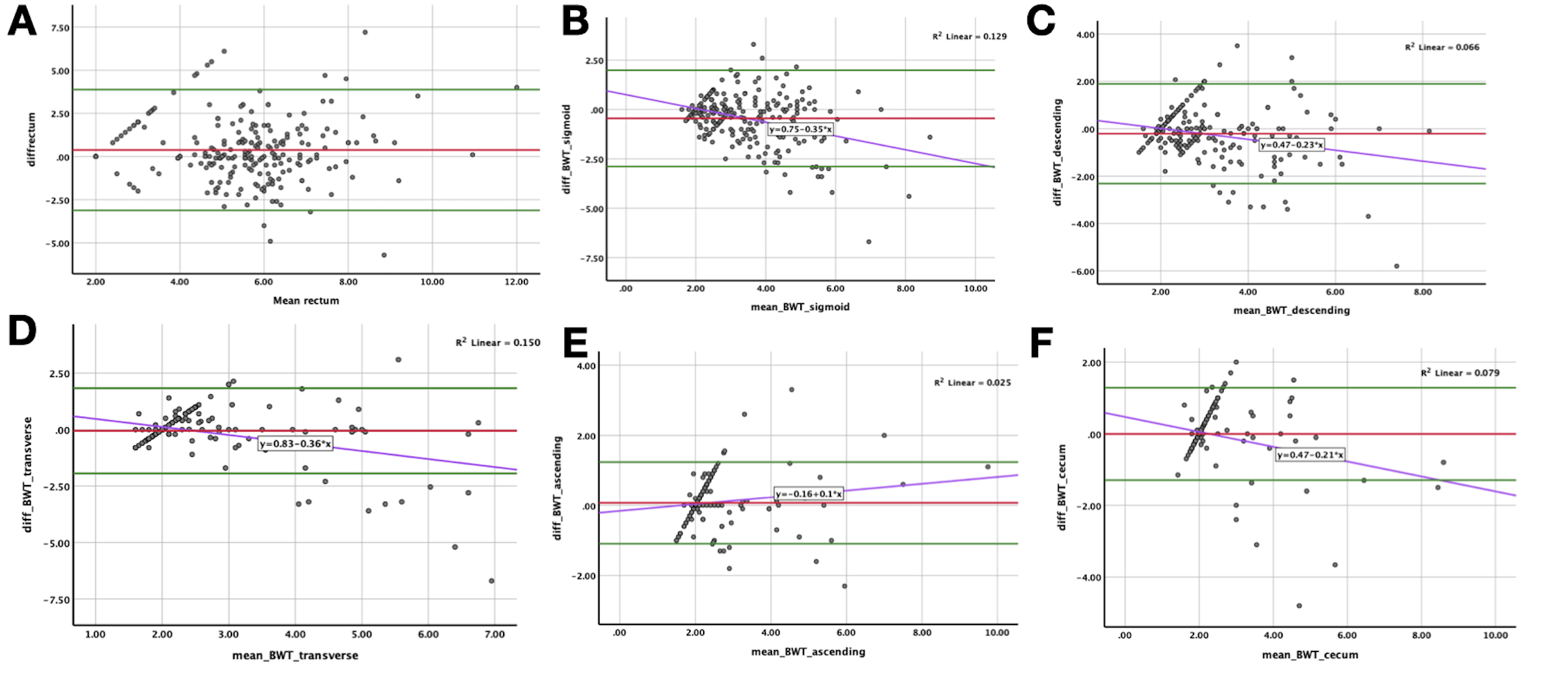


**Supplementary Figure 4.** Correlation between mid-end and high-end Milan Ultrasound Criteria (MUC) scores across colonic segments in ulcerative colitis. Scatter plots for caecum (A), ascending colon (B), transverse colon (C), descending colon (D), sigmoid (E), and rectum (F) show consistent positive correlation across platforms.


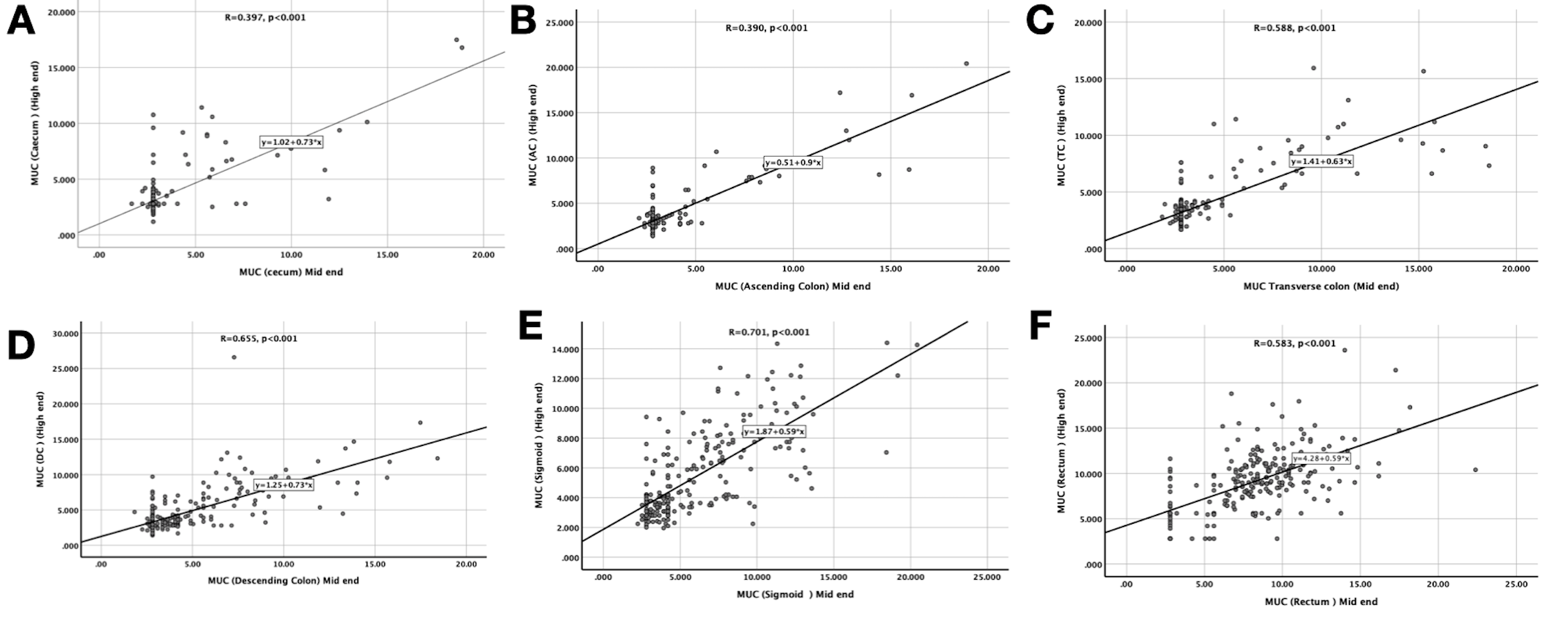


**Supplementary Figure 5.** Correlation of overall and rectum-excluded Milan Ultrasound Criteria (MUC) scores between mid-end and high-end systems in ulcerative colitis. Panels display strong correlation for rectum-excluded MUC (A) and overall MUC (B), supporting interchangeability of platforms for severity quantification.
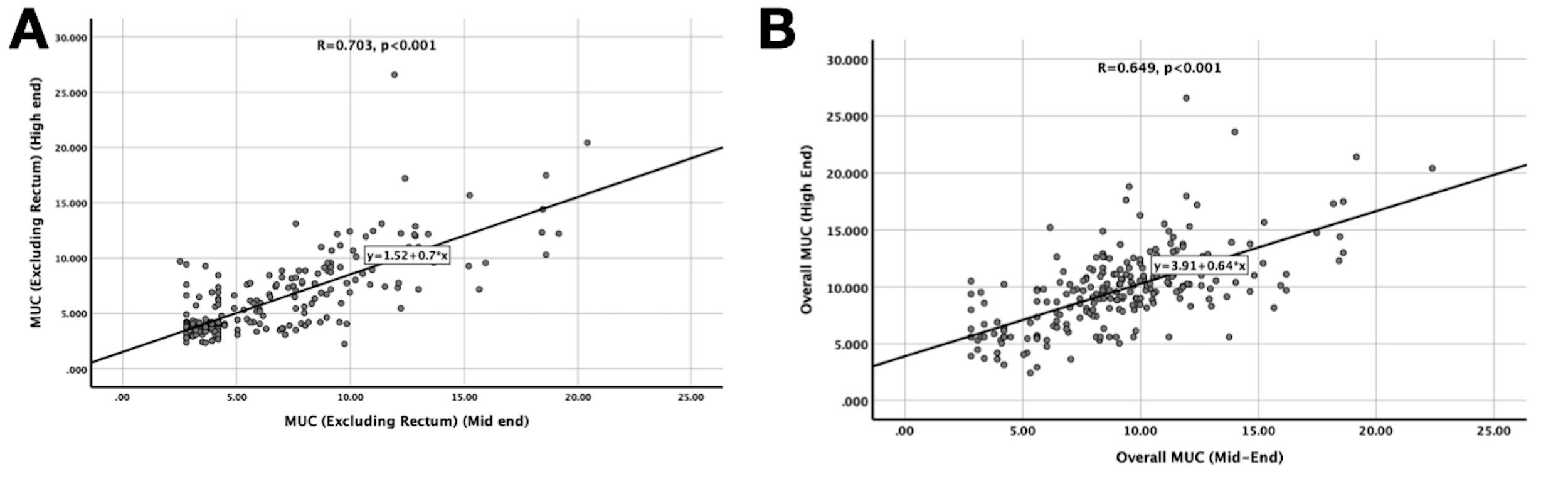


**Supplementary Figure 6.** Bland–Altman agreement plots for segmental Milan Ultrasound Criteria (MUC) scores measured with mid-end and high-end ultrasound in ulcerative colitis. Agreement for rectum (A), sigmoid (B), descending (C), transverse (D), ascending (E), and caecum (F) shows low mean difference and symmetric distribution across the measurement range.
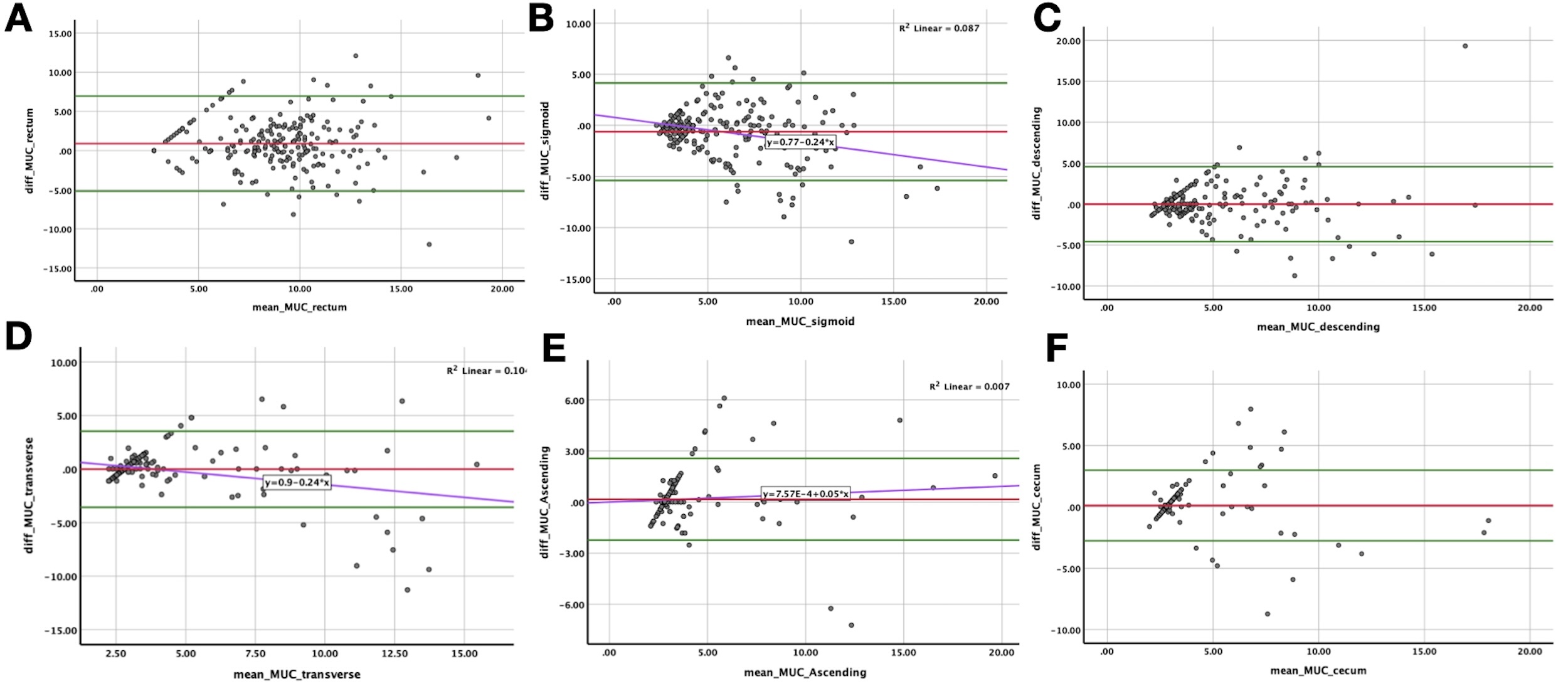


**Supplementary Figure 7.** Bland–Altman plot for overall Milan Ultrasound Criteria (MUC) scores in ulcerative colitis. The plot demonstrates minimal systematic bias and narrow limits of agreement between mid-end and high-end ultrasound systems for global disease activity scoring.


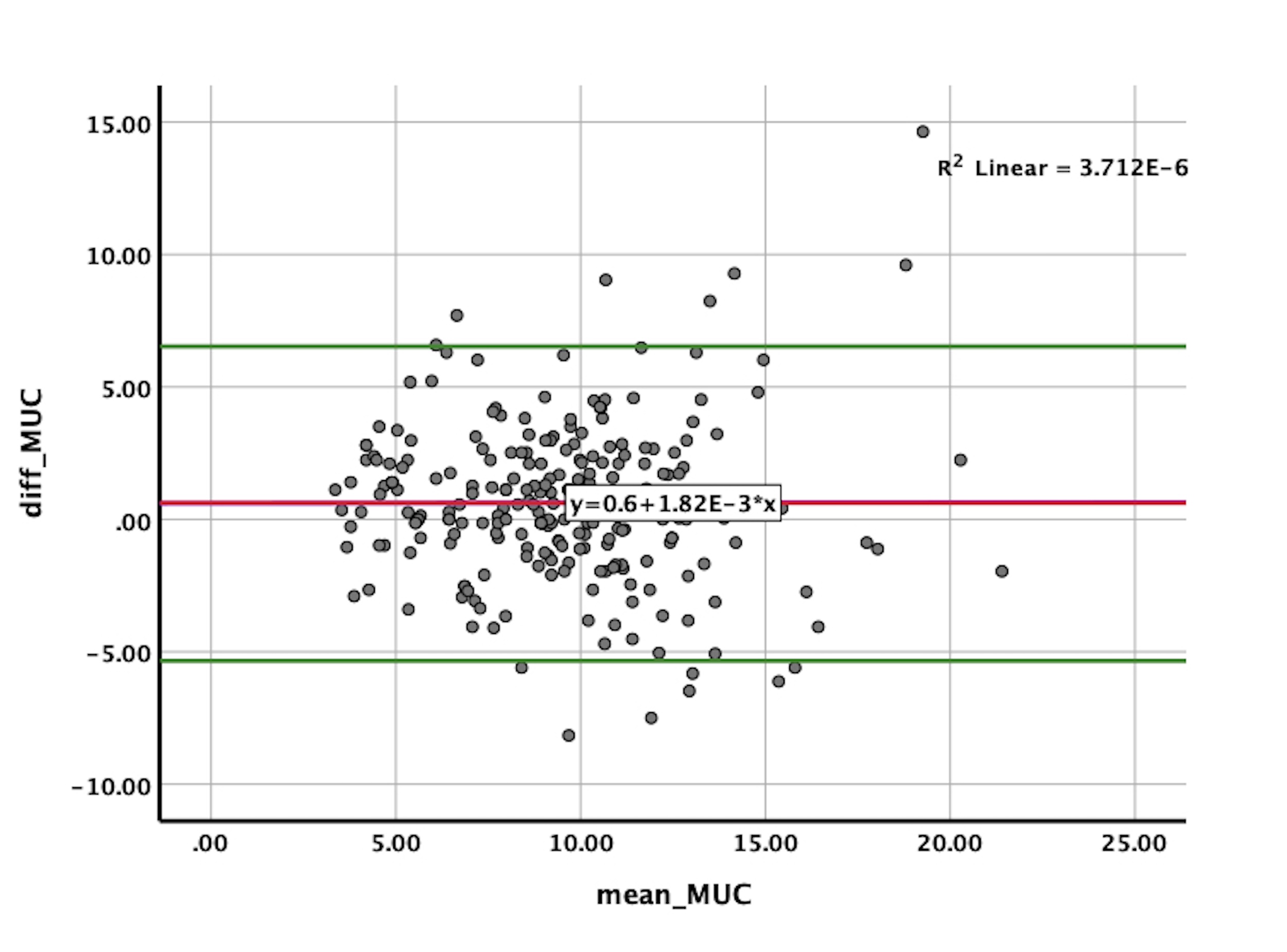


**Supplementary Figure 8.** Crohn’s disease: Segment-wise correlation of bowel wall thickness (BWT) between mid-end and high-end IUS systems. Scatter plots (A–G) represent terminal ileum, caecum, ascending colon, transverse colon, descending colon, sigmoid, and rectum. Linear regression demonstrates strong correlation across all CD segments, including the terminal ileum.


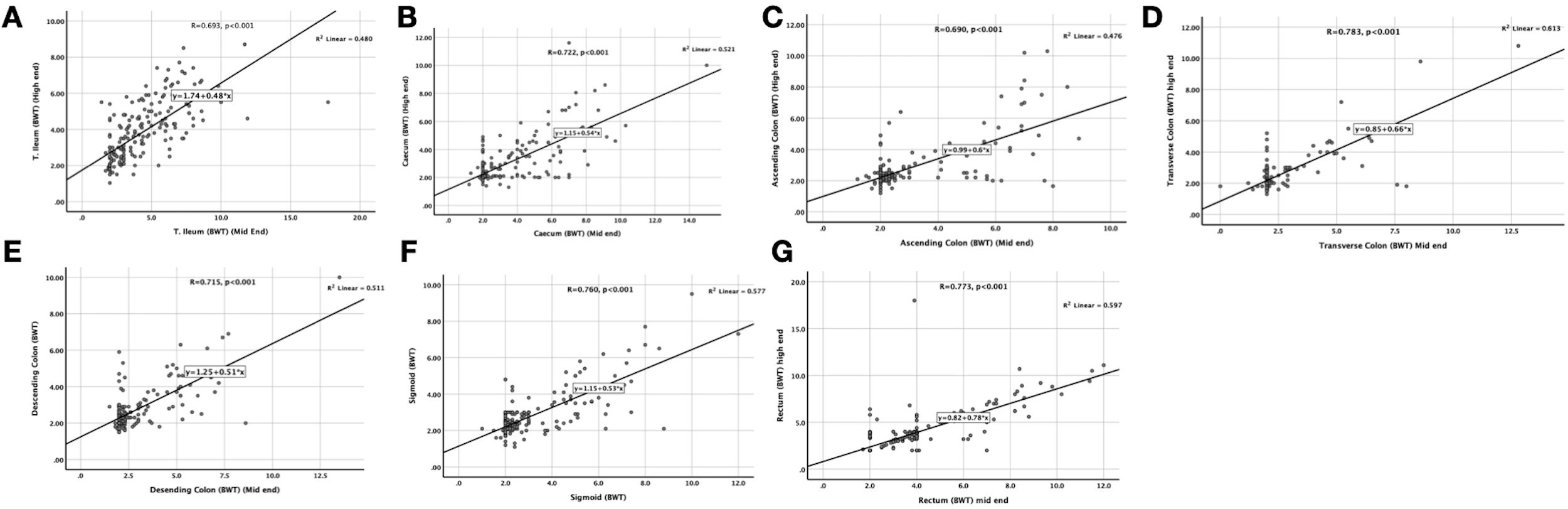


**Supplementary Figure 9.** Crohn’s disease: Bland–Altman agreement analyses for BWT. Panels (A–G) show mean–difference plots for terminal ileum, caecum, ascending colon, transverse colon, descending colon, sigmoid, and rectum. Limits of agreement indicate good concordance, with small mean differences
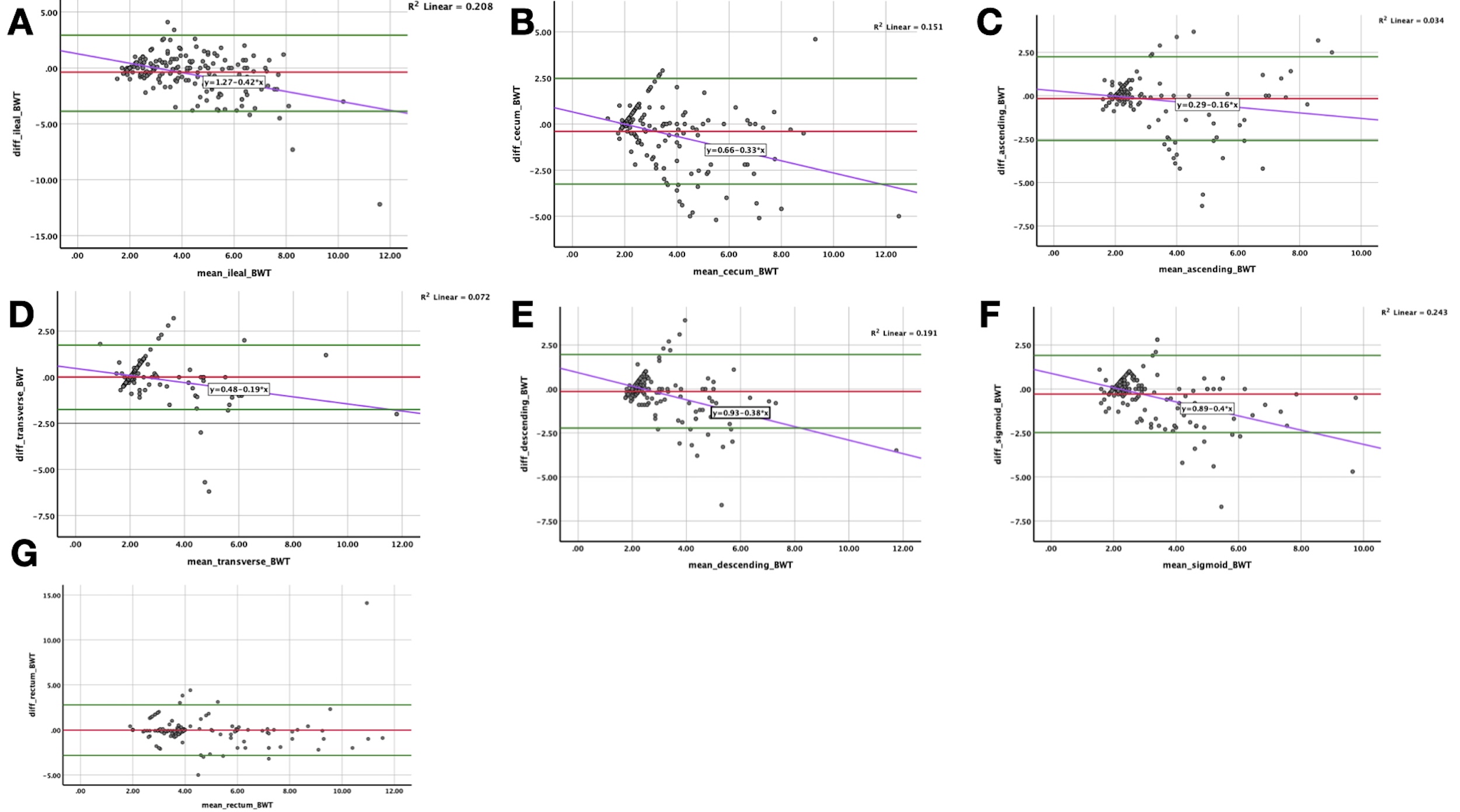


**Supplementary Figure 10**. Crohn’s disease: Segment-wise correlation of IBUS-SAS between systems. Panels (A–F) include terminal ileum, right colon (caecum/ascending), transverse colon, left colon (descending/sigmoid), rectum, and overall IBUS-SAS (excluding rectum), with an additional panel (G) for overall IBUS-SAS including rectum. Strong correlations were observed in all compartments.
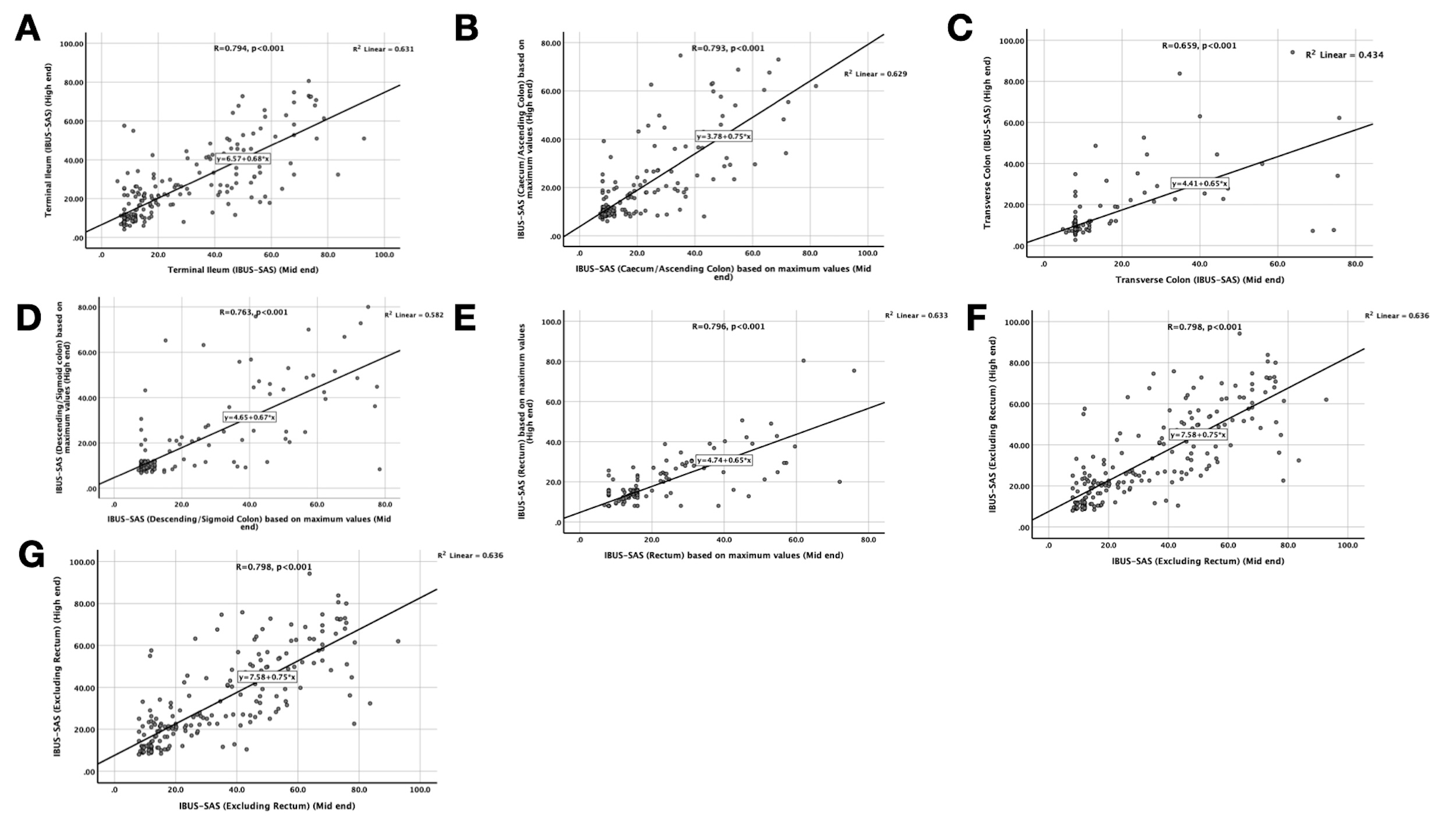


**Supplementary Figure 11**. Crohn’s disease: Bland–Altman plots for IBUS-SAS.Panels (A–F) depict agreement for terminal ileum, right colon, transverse colon, left colon, and rectum. Panel G: Overall IBUS-SAS excluding rectum. Panel H (F in your file): Overall IBUS-SAS including rectum. Mean differences were small with symmetrical scatter, supporting interchangeability of platforms.
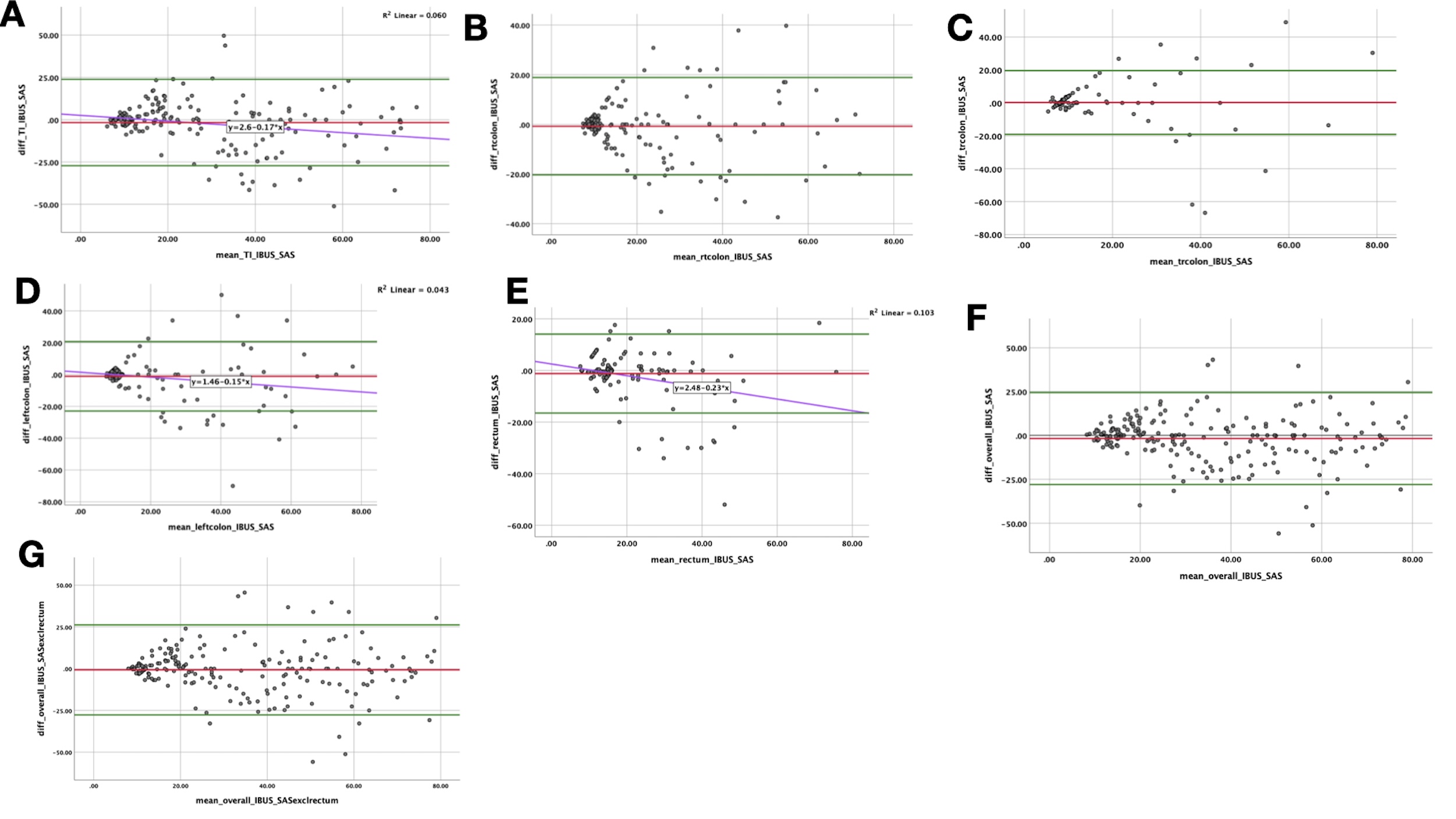


**Supplementary Figure 12.** Decision curve analysis for A) ulcerative colitis and B) Crohn’s disease comparing mid-end versus high-end ultrasound models. Decision curves demonstrate the net benefit of MUC-based mid-end and high-end ultrasound models across threshold probabilities for predicting endoscopically active ulcerative colitis. Both models outperform the "treat-none" strategy across all thresholds and remain superior to the "treat-all" strategy except at very high thresholds. The curves show near-overlapping trajectories, indicating minimal difference in clinical utility between platforms.


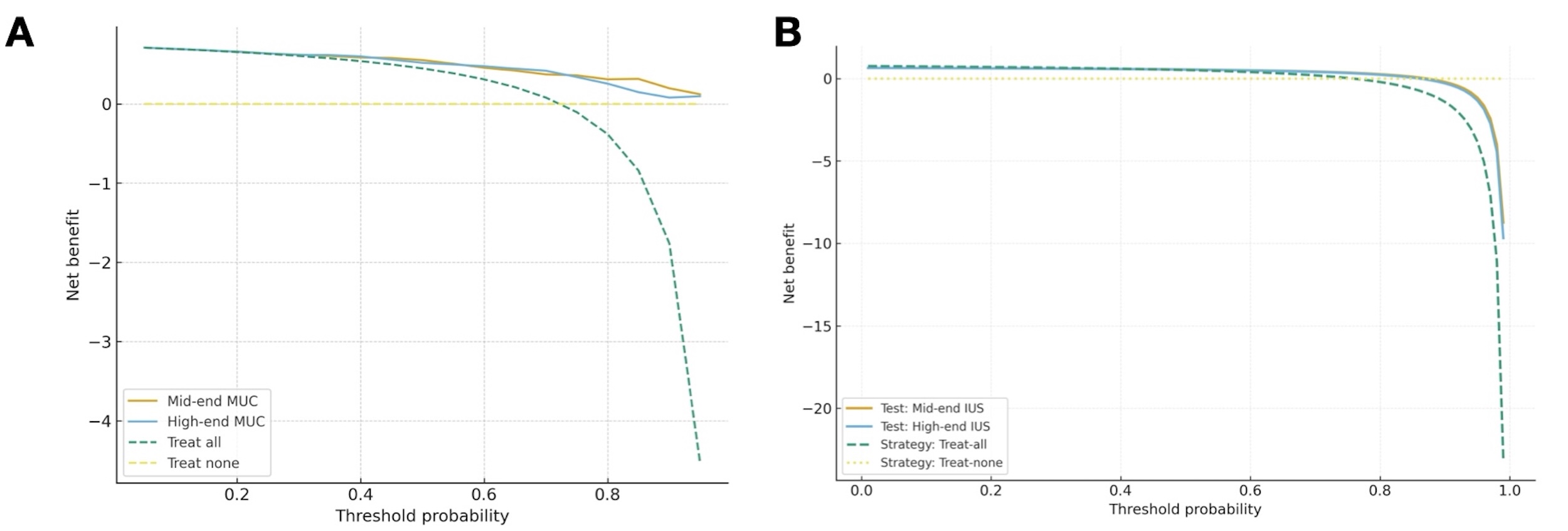


# Supplementary Table 1 : Technical Comparison of Mid-Range and High-End Ultrasound Platforms Used for Intestinal Ultrasound

| **Technical Domain** | **Mid-Range Ultrasound Platform** | **High-End Ultrasound Platform** |
| --- | --- | --- |
| **Processing Capacity** | Standard computational capability appropriate for real-time bowel imaging; limited advanced dynamic post-processing tools. | Markedly higher processing performance (often described in billion operations per second – BOPS), enabling advanced real-time rendering, motion tracking, and quantitative analysis. |
| **Transducer Technology** | Commonly uses piezoelectric ceramic transducer elements. | Often employs single-crystal transducer technology, improving signal efficiency and uniformity. |
| **Spatial Resolution** | Moderate spatial sharpness adequate for routine wall thickness assessment. | Higher spatial resolution supported by enhanced beamforming architecture. |
| **Layer Detail Visualization** | Capable of visualizing bowel wall layers, though subtle stratification may require careful optimization. | Improved visualization of fine mural architecture due to refined signal processing algorithms. |
| **Contrast Optimization** | Basic grayscale and Doppler optimization functions. | Advanced image-enhancement capabilities, including refined speckle suppression and edge enhancement. |
| **Depth Penetration** | Sufficient penetration for ileocolonic evaluation; clarity may decrease at greater depths. | Improved penetration while preserving image sharpness across deeper structures. |
| **Resolution Uniformity with Depth** | Image consistency may vary depending on depth. | More uniform image quality maintained throughout the depth field. |
| **Frequency Bandwidth** | Operates within narrower linear probe frequency ranges (e.g., 4–9 MHz or 5–12 MHz). | Provides broader frequency bandwidth options (e.g., 2–14 MHz), allowing better trade-off between penetration and resolution. |
| **Workflow Efficiency** | May require longer acquisition time and greater operator experience for optimal bowel assessment. | Often includes dedicated bowel presets and enhanced Doppler sensitivity, potentially facilitating faster acquisition and ease of use. |
| **Capital Cost** | Typically utilizes existing installed systems without major additional capital expenditure. | Requires significant new procurement investment, depending on configuration. |

Supplementary table 2. Definitions of diagnostic, agreement, and scoring metrics used in the HUMID study

| **Term** | **Definition Used in This Study** | **Explanation** |
| --- | --- | --- |
| **True Positive (TP)** | Ultrasound positive AND ileocolonoscopy active | Correct identification of active disease |
| **False Positive (FP)** | Ultrasound positive AND ileocolonoscopy inactive | Incorrect identification of active disease |
| **True Negative (TN)** | Ultrasound negative AND ileocolonoscopy inactive | Correct exclusion of disease |
| **False Negative (FN)** | Ultrasound negative AND ileocolonoscopy active | Missed active disease |
| **Sensitivity** | TP / (TP + FN) | Proportion of active disease correctly detected |
| **Specificity** | TN / (TN + FP) | Proportion of inactive disease correctly identified |
| **Positive Predictive Value (PPV)** | TP / (TP + FP) | Probability that ultrasound-positive cases are truly active |
| **Negative Predictive Value (NPV)** | TN / (TN + FN) | Probability that ultrasound-negative cases are truly inactive |
| **Diagnostic Accuracy** | (TP + TN) / (TP + TN + FP + FN) | Overall proportion correctly classified |
| **Absolute Difference (Δ)** | Metric_high-end − Metric_mid-end | Difference in performance between systems |
| **McNemar Test** | Paired comparison using discordant classifications | Tests whether paired proportions differ |
| **ROC Curve** | Plot of sensitivity vs 1−specificity across thresholds | Evaluates discrimination of continuous scores |
| **Area Under the Curve (AUC)** | Integral of the receiver operating characteristic (ROC) curve (range 0.5–1.0) | Higher values indicate better discrimination |
| **Spearman Correlation (ρ)** | Rank-based correlation coefficient | Measures monotonic association between ultrasound and endoscopy |
| **Bland–Altman Mean Difference** | Mean (High-end − Mid-end) measurement | Estimates systematic bias |
| **Bland–Altman Limits of Agreement** | Mean difference ± 1.96 × standard deciation of differences | Range within which most inter-system differences lie |
| **Concordance Correlation Coefficient (CCC)** | Agreement measure combining precision and bias | Values closer to 1 indicate stronger concordance |
| **Clinically Acceptable Difference** | Absolute inter-system BWT difference ≤1 mm | Predefined threshold for interchangeability |
| **Diagnostic Odds Ratio (DOR)** | (TP × TN) / (FP × FN) | Summary measure of diagnostic discrimination |
| **Net Reclassification Improvement (NRI)** | Net proportion of correctly reclassified cases when switching systems | Quantifies management impact |
| **Decision Curve Analysis (DCA)** | Method estimating net clinical benefit across threshold probabilities | Assesses real-world clinical usefulness |
| **Net Benefit** | (TP/n) − (FP/n) × (pt/(1−pt)) | Clinical utility at a chosen threshold probability |
| **Ulcerative Colitis Endoscopic Index of Severity (UCEIS)** | Vascular pattern (0–2) + Bleeding (0–3) + Erosions/Ulcers (0–3); total 0–8; Active ≥2 | Validated endoscopic severity index for UC |
| **Simple Endoscopic Score for Crohn’s Disease (SES-CD)** | Ulcer size + Ulcerated surface + Affected surface + Narrowing (each 0–3 per segment); Active ≥3 | Standard segmental endoscopic activity score for CD |
| **Milan Ultrasound Criteria (MUC)** | MUC = (1.4 × Bowel Wall Thickness: BWT in mm) + (2 × Color Doppler signal: CDS); Active >6.2 | Validated sonographic activity index for UC |
| **International Bowel Ultrasound Simplified Activity Score (IBUS-SAS)** | IBUS-SAS = (4 × BWT) + (15 × inflammatory-fat) + (7 × CDS) + (4 × bowel wall stratification:BWS) | Composite sonographic activity index for CD |
| **Modified Limberg Score** | Doppler vascularity grading (0–3); ≥1 considered hypervascular | Assesses bowel wall vascular signal |
| **Ultrasound-defined Active Disease** | BWT >3 mm (>4 mm rectum TPUS) and/or Doppler signal and/or loss of stratification and/or inflammatory fat | Binary operational definition used for diagnostic accuracy analysis |

**Supplementary Table 3.** Segment-wise continuous intestinal ultrasound and endoscopic activity measurements in ulcerative colitis. This table summarizes paired quantitative metrics obtained using mid-end and high-end intestinal ultrasound systems across all colonic segments in patients with ulcerative colitis

| **Variables** | **N** | **Median** | **Minimum** | **Maximum** |
| --- | --- | --- | --- | --- |
|  | Valid |  |  |  |
| **Rectum (BWT) (Mid End Avg.)** | 238 | 5.5 | 2 | 11.7 |
| **Rectum (BWT) (High End) (TPUS Avg.)** | 239 | 5.6 | 2 | 14 |
| **Sigmoid (BWT) (Mid end)** | 239 | 3 | 1.6 | 10.3 |
| **Sigmoid (BWT) (High end)** | 239 | 2.9 | 1.4 | 8 |
| **Desending Colon (BWT)** | 239 | 2.5 | 1.3 | 10.3 |
| **Desending Colon (BWT) (High end)** | 239 | 2.4 | 1 | 8.1 |
| **D.colon UCEIS** | 238 | 0 | 0 | 7 |
| **Rectum**  **UCEIS** | 239 | 2 | 0 | 7 |
| **Sigmoid UCEIS** | 239 | 0 | 0 | 7 |
| **Transverse Colon (BWT) (Mid end)** | 239 | 2 | 1.3 | 10.3 |
| **Transverse Colon (BWT) (High end)** | 239 | 2 | 1.2 | 7.1 |
| **T.colon UCEIS** | 238 | 0 | 0 | 6 |
| **Ascending Colon (BWT) (Mid end)** | 239 | 2 | 1.5 | 9.2 |
| **A.Colon UCEIS** | 235 | 0 | 0 | 6 |
| **Ascending Colon (BWT) (High end)** | 239 | 2 | 1 | 10.3 |
| **Caecum (BWT) (Mid end)** | 239 | 2 | 1.2 | 9.2 |
| **Caecum UCEIS** | 235 | 0 | 0 | 6 |
| **Caecum (BWT)** | 239 | 2 | 0.85 | 8.2 |
| **MUC (Rectum ) Mid end** | 239 | 8.26 | 2.8 | 22.38 |
| **MUC (Sigmoid ) Mid end** | 239 | 4.2 | 2.24 | 20.42 |
| **MUC (Descending Colon) Mid end** | 239 | 3.5 | 1.82 | 18.42 |
| **MUC Transverse colon (Mid end)** | 239 | 2.8 | 1.82 | 18.6 |
| **MUC (Ascending Colon) Mid end** | 239 | 2.8 | 2.1 | 18.88 |
| **MUC (cecum) Mid end** | 239 | 2.8 | 1.68 | 18.88 |
| **Overall MUC (Mid-End)** | 239 | 9 | 2.8 | 22.38 |
| **MUC (Excluding Rectum) (Mid end)** | 239 | 4.48 | 2.52 | 20.42 |
| **MUC (Rectum ) (High end)** | 239 | 9 | 2.8 | 23.6 |
| **MUC (Sigmoid ) (High end)** | 239 | 4.2 | 1.96 | 14.4 |
| **MUC (DC ) (High end)** | 239 | 3.36 | 1.4 | 26.58 |
| **MUC (TC ) (High end)** | 239 | 2.8 | 1.68 | 15.94 |
| **MUC (AC ) (High end)** | 239 | 2.8 | 1.4 | 20.42 |
| **MUC (Caecum ) (High end)** | 239 | 2.8 | 1.19 | 17.48 |
| **Overall MUC (High End)** | 239 | 9.6 | 2.422 | 26.58 |
| **MUC (Excluding Rectum) (High end)** | 239 | 4.2 | 2.24 | 26.58 |
| **UCEIS** | 239 | 3 | 0 | 7 |

**Supplementary Table 4.** Segment-wise continuous intestinal ultrasound and endoscopic activity measurements in Crohn’s disease.

| **Variables** | **N** | **Median** | **Minimum** | **Maximum** |
| --- | --- | --- | --- | --- |
| **T. Ileum (BWT) (Mid End)** | 211 | 3.3 | 1.4 | 17.7 |
| **T. Ileum (BWT) (High end)** | 211 | 3.5 | 1.03 | 8.7 |
| **Caecum (BWT) (Mid end)** | 211 | 2.1 | 1.2 | 15 |
| **Caecum (IBUS-SAS) (Mid end)** | 211 | 8.4 | 4.8 | 82 |
| **Ascending Colon (BWT) (Mid end)** | 210 | 2.2 | 1.2 | 8.9 |
| **Ascending colon (IBUS-SAS) (Mid end)** | 211 | 8.8 | 4.8 | 71.6 |
| **IBUS-SAS (Caecum)(High end)** | 211 | 8.8 | 5.2 | 73 |
| **Ascending Colon (BWT) (High end)** | 211 | 2.3 | 1.2 | 10.3 |
| **IBUS-SAS (Ascending Colon)(High end)** | 211 | 9.2 | 0 | 74.68 |
| **Transverse Colon (BWT)** | 211 | 2 | 0 | 12.8 |
| **Transverse Colon (BWT)** | 211 | 2 | 1.3 | 10.8 |
| **Desending Colon (BWT) (Mid end)** | 211 | 2.2 | 1.5 | 13.5 |
| **Sigmoid (BWT)** | 211 | 2.3 | 1 | 12 |
| **Sigmoid (CDS)** | 211 | 0 | 0 | 3 |
| **IBUS-SAS (Descending Colon) based on maximum values (Mid end)** | 211 | 8.8 | 0 | 78.4 |
| **IBUS-SAS (Sigmoid Colon) based on maximum values (Mid end)** | 211 | 9.2 | 0 | 77 |
| **Descending Colon (BWT)** | 211 | 2.3 | 1.5 | 10 |
| **IBUS-SAS (Descending Colon)(High end)** | 211 | 9.2 | 6 | 80 |
| **Sigmoid (BWT)** | 211 | 2.4 | 1.1 | 9.5 |
| **IBUS-SAS (Sigmoid) (High end)** | 211 | 9.6 | 4.4 | 75.8 |
| **Rectum (BWT)** | 211 | 3.4 | 1.7 | 12 |
| **Rectum (BWT)** | 211 | 3.3 | 2 | 18 |
| **Terminal Ileum (IBUS-SAS) (Mid end)** | 210 | 14.8 | 5.6 | 92.8 |
| **IBUS-SAS (Right colon) (Mid end)** | 210 | 10 | 7.2 | 82 |
| **Transverse Colon (IBUS-SAS) (Mid end)** | 211 | 8 | 4.8 | 75.8 |
| **IBUS-SAS (Descending/Sigmoid Colon) based on maximum values (Mid end)** | 211 | 9.2 | 7.2 | 78.4 |
| **IBUS-SAS (Rectum) based on maximum values (Mid end)** | 211 | 13.6 | 6.8 | 76 |
| **IBUS-SAS(Mid end Ultrasound)** | 211 | 29.6 | 8 | 92.8 |
| **IBUS-SAS (Excluding Rectum) (Mid end)** | 211 | 27 | 8 | 92.8 |
| **Terminal Ileum (IBUS-SAS) (High end)** | 211 | 18.8 | 4.12 | 80.6 |
| **IBUS-SAS (Right colon)(High end)** | 211 | 10 | 6.04 | 74.68 |
| **Transverse Colon (IBUS-SAS) (High end)** | 211 | 8 | 2.8 | 94.2 |
| **IBUS-SAS (Descending/Sigmoid colon) based on maximum values (High end)** | 211 | 10 | 6.8 | 80 |
| **IBUS-SAS (Rectum) based on maximum values (High end)** | 211 | 13.2 | 8 | 80.4 |
| **IBUS-SAS(High-end Ultrasound)** | 211 | 27 | 0 | 94.2 |
| **IBUS-SAS (Excluding Rectum) (High end)** | 211 | 26.2 | 8 | 94.2 |
| **Total SES-CD** | 211 | 5 | 0 | 30 |

**Supplementary Table 5.** Area under the ROC curve (AUC) for bowel wall thickness (BWT) and Milan Ultrasound Criteria (MUC) using mid-end and high-end ultrasound systems across colonic segments in ulcerative colitis. AUC values with corresponding 95% confidence intervals are presented for each colonic segment, along with overall MUC performance and MUC excluding the rectum.

| **Area Under the ROC Curve** |  |  |  |
| --- | --- | --- | --- |
| **Test Result Variable(s)** | **Area** | **Asymptotic 95% Confidence Interval** |  |
|  |  | **Lower Bound** | **Upper Bound** |
| **Rectum (BWT) (Mid End)** | 0.770 | 0.701 | 0.838 |
| **Rectum (BWT) (High End)** | 0.721 | 0.646 | 0.796 |
| **MUC (Rectum ) Mid end** | 0.813 | 0.754 | 0.872 |
| **MUC (Rectum ) (High end)** | 0.803 | 0.743 | 0.864 |
| **Sigmoid (BWT) (Mid end)** | 0.850 | 0.800 | 0.900 |
| **MUC (Sigmoid ) Mid end** | 0.848 | 0.798 | 0.897 |
| **Sigmoid (BWT) (High end)** | 0.831 | 0.777 | 0.885 |
| **MUC (Sigmoid ) (High end)** | 0.851 | 0.801 | 0.902 |
| **Desending Colon (BWT)** | 0.804 | 0.738 | 0.871 |
| **MUC (Descending Colon) Mid end** | 0.819 | 0.752 | 0.885 |
| **Desending Colon (BWT) (High end)** | 0.839 | 0.784 | 0.893 |
| **MUC (DC ) (High end)** | 0.857 | 0.804 | 0.910 |
| **Transverse Colon (BWT) (Mid end)** | 0.892 | 0.830 | 0.954 |
| **Transverse Colon (BWT) (High end)** | 0.913 | 0.858 | 0.967 |
| **MUC Transverse colon (Mid end)** | 0.894 | 0.832 | 0.956 |
| **MUC (TC ) (High end)** | 0.925 | 0.871 | 0.978 |
| **Ascending Colon (BWT) (Mid end)** | 0.823 | 0.715 | 0.931 |
| **Ascending Colon (BWT) (High end)** | 0.881 | 0.787 | 0.974 |
| **MUC (Ascending Colon) Mid end** | 0.823 | 0.715 | 0.931 |
| **MUC (AC ) (High end)** | 0.891 | 0.798 | 0.984 |
| **Caecum (BWT) (Mid end)** | 0.813 | 0.715 | 0.911 |
| **Caecum (BWT)** | 0.794 | 0.660 | 0.927 |
| **MUC (cecum) Mid end** | 0.811 | 0.713 | 0.908 |
| **MUC (Caecum ) (High end)** | 0.798 | 0.665 | 0.932 |
| **Overall MUC (Mid-End)** | 0.851 | 0.799 | 0.903 |
| **MUC (Excluding Rectum) (Mid end)** | 0.766 | 0.706 | 0.825 |
| **Overall MUC (High End)** | 0.831 | 0.772 | 0.891 |

**Supplementary Table 6.** Area under the ROC curve (AUC) for bowel wall thickness (BWT) and the International Bowel Ultrasound Segmental Activity Score (IBUS-SAS) using mid-end and high-end ultrasound systems across ileocolonic segments in Crohn’s disease.

| Area Under the ROC Curve |  |  |  |
| --- | --- | --- | --- |
| Test Result Variable(s) | Area | Asymptotic 95% Confidence Interval |  |
|  |  | Lower Bound | Upper Bound |
| T. Ileum (BWT) (Mid End) | 0.889 | 0.843 | 0.935 |
| T. Ileum (BWT) (High end) | 0.880 | 0.831 | 0.930 |
| Terminal Ileum (IBUS-SAS) (Mid end) | 0.894 | 0.849 | 0.939 |
| Terminal Ileum (IBUS-SAS) (High end) | 0.896 | 0.852 | 0.941 |
| IBUS-SAS (Caecum/Ascending Colon) based on maximum values (Mid end) | 0.810 | 0.726 | 0.894 |
| IBUS-SAS (Caecum/Ascending Colon) based on maximum values (High end) | 0.856 | 0.778 | 0.934 |
| Transverse Colon (BWT) Mid end | 0.859 | 0.776 | 0.941 |
| Transverse Colon (BWT) high end | 0.832 | 0.732 | 0.931 |
| Transverse Colon (IBUS-SAS) (Mid end) | 0.860 | 0.776 | 0.943 |
| Transverse Colon (IBUS-SAS) (High end) | 0.837 | 0.738 | 0.937 |
| IBUS-SAS (Descending/Sigmoid Colon) based on maximum values (Mid end) | 0.848 | 0.774 | 0.923 |
| IBUS-SAS (Descending/Sigmoid colon) based on maximum values (High end) | 0.890 | 0.826 | 0.953 |
| Rectum (BWT) mid end | 0.850 | 0.761 | 0.940 |
| Rectum (BWT) high end | 0.839 | 0.747 | 0.932 |
| IBUS-SAS (Rectum) based on maximum values (Mid end) | 0.873 | 0.789 | 0.956 |
| IBUS-SAS (Rectum) based on maximum values (High end) | 0.852 | 0.759 | 0.945 |
| IBUS-SAS(Mid end Ultrasound) | 0.821 | 0.764 | 0.879 |
| IBUS-SAS(High-end Ultrasound) | 0.825 | 0.764 | 0.886 |
| IBUS-SAS (Excluding Rectum) (Mid end) | 0.818 | 0.759 | 0.877 |
| IBUS-SAS (Excluding Rectum) (High end) | 0.831 | 0.771 | 0.891 |

**Supplementary Table 7.**Segment-wise concordance, precision, and accuracy between high-end and mid-end ultrasound systems for bowel wall thickness measurements in ulcerative colitis. This table presents Lin’s Concordance Correlation Coefficient (CCC), its precision component (Pearson correlation, *r*), and accuracy component (bias correction factor, *Cᵦ*) for bowel wall thickness across all colonic segments.

| **Segment** | **Precision_r** | **Accuracy_Cb** | **CCC** |
| --- | --- | --- | --- |
| **Rectum BWT** | 0.553 | 0.981 | 0.5425 |
| **Sigmoid BWT** | 0.644 | 0.913 | 0.588 |
| **Descending BWT** | 0.681 | 0.97 | 0.661 |
| **Transverse BWT** | 0.683 | 0.955 | 0.652 |
| **Ascending BWT** | 0.83 | 0.995 | 0.826 |
| **Caecum BWT** | 0.773 | 0.983 | 0.76 |

**Supplementary Table 8.** Segment-wise concordance, precision, and accuracy between high-end and mid-end ultrasound systems for bowel wall thickness in Crohn’s disease. This table summarizes Lin’s CCC, precision (Pearson *r*), and accuracy (bias correction factor *Cᵦ*) for bowel wall thickness across segments.

| **Segment** | **Accuracy_r** | **Precision_Cb** | **CCC** |
| --- | --- | --- | --- |
| **Terminal ileum** | 0.693 | 0.774 | 0.536 |
| **Cecum** | 0.722 | 0.838 | 0.605 |
| **Ascending colon** | 0.69 | 0.959 | 0.662 |
| **Transverse colon** | 0.783 | 0.943 | 0.738 |
| **Descending colon** | 0.715 | 0.808 | 0.578 |
| **Sigmoid colon** | 0.76 | 0.774 | 0.588 |
| **Rectum** | 0.773 | 0.9999 | 0.773 |

**Supplementary Table 9**.Clinical acceptability of bowel wall thickness (BWT) measurements between high-end and mid-end ultrasound systems in ulcerative colitis. This table reports the proportion of paired BWT measurements falling within the predefined clinical acceptability threshold of ±1 mm across all colonic segments.

| **Segment** | **Mean Diff (mm)** | **SD (mm)** | **LOA Lower (mm)** | **LOA Upper (mm)** | **% Acceptable (≤1 mm)** | **CCC** |
| --- | --- | --- | --- | --- | --- | --- |
| **Cecum BWT** | -0.005 | 0.65797 | -1.2946212 | 1.2846212 | 92.5 | 0.76 |
| **Ascending BWT** | 0.0704 | 0.59372 | -1.0932912 | 1.2340912 | 92.5 | 0.826 |
| **Transverse BWT** | -0.0171 | 2.3301 | -4.584096 | 4.549896 | 90 | 0.652 |
| **Descending BWT** | -0.2095 | 1.07354 | -2.3136384 | 1.8946384 | 79.5 | 0.661 |
| **Sigmoid BWT** | -0.4499 | 1.24406 | -2.8882576 | 1.9884576 | 69.5 | 0.588 |
| **Rectum BWT** | -0.61993 | 2.43223 | -5.3871008 | 4.1472408 | 54.2 | 0.5425 |

**Supplementary Table 10.** Clinical acceptability of bowel wall thickness (BWT) measurements between high-end and mid-end ultrasound systems in Crohn’s disease. This table summarizes the percentage of paired BWT measurements within ±1 mm for segments.

| **Segment** | **Mean Diff (mm)** | **SD (mm)** | **LOA Lower (mm)** | **LOA Upper (mm)** | **Acceptability (%)** | **CCC** |
| --- | --- | --- | --- | --- | --- | --- |
| **Terminal ileum** | -0.3779 | 1.68629 | -3.683 | 2.9272 | 87.2 | 0.536 |
| **Cecum** | -0.005 | 0.65797 | -1.2956 | 1.2856 | 93.8 | 0.605 |
| **Ascending colon** | -0.2865 | 1.11991 | -2.48149 | 1.90849 | 95.2 | 0.662 |
| **Transverse colon** | -0.0064 | 0.89106 | -1.75288 | 1.74008 | 95.7 | 0.738 |
| **Descending colon** | -0.1373 | 1.06727 | -2.22914 | 1.95454 | 95.7 | 0.578 |
| **Sigmoid colon** | -0.1642 | 1.23105 | -2.57706 | 2.24866 | 97.16 | 0.588 |
| **Rectum** | -0.7153 | 13.75256 | -27.6523 | 26.2217 | 90.52 | 0.773 |

**Supplementary Table 11**. Decision curve analysis for ulcerative colitis: net benefit of mid-end and high-end ultrasound models across threshold probabilities. This table summarizes the net benefit of mid-end and high-end intestinal ultrasound (IUS) models for predicting endoscopically active ulcerative colitis across clinically relevant threshold probabilities. The performance of each model is benchmarked against the "treat-all" and "treat-none" reference strategies. Higher net benefit values indicate superior clinical utility at the corresponding threshold.

| **Threshold** | **TP_Mid** | **FP_Mid** | **NetBenefit_Mid** | **TP_High** | **FP_High** | **NetBenefit_High** | **NetBenefit_TreatAll** | **NetBenefit_TreatNone** |
| --- | --- | --- | --- | --- | --- | --- | --- | --- |
| 0.05 | 173 | 66 | 0.71 | 173 | 66 | 0.71 | 0.71 | 0 |
| 0.1 | 173 | 66 | 0.69 | 173 | 65 | 0.69 | 0.69 | 0 |
| 0.15 | 172 | 60 | 0.68 | 173 | 63 | 0.68 | 0.68 | 0 |
| 0.2 | 171 | 53 | 0.66 | 172 | 59 | 0.66 | 0.65 | 0 |
| 0.25 | 167 | 44 | 0.64 | 171 | 57 | 0.64 | 0.63 | 0 |
| 0.3 | 167 | 43 | 0.62 | 171 | 54 | 0.62 | 0.61 | 0 |
| 0.35 | 167 | 42 | 0.60 | 165 | 33 | 0.62 | 0.58 | 0 |
| 0.4 | 166 | 39 | 0.59 | 164 | 31 | 0.60 | 0.54 | 0 |
| 0.45 | 163 | 30 | 0.58 | 159 | 31 | 0.56 | 0.50 | 0 |
| 0.5 | 160 | 28 | 0.55 | 154 | 30 | 0.52 | 0.45 | 0 |
| 0.55 | 153 | 26 | 0.51 | 153 | 28 | 0.50 | 0.39 | 0 |
| 0.6 | 148 | 26 | 0.46 | 151 | 25 | 0.47 | 0.31 | 0 |
| 0.65 | 145 | 24 | 0.42 | 147 | 22 | 0.44 | 0.21 | 0 |
| 0.7 | 138 | 21 | 0.37 | 142 | 18 | 0.42 | 0.08 | 0 |
| 0.75 | 131 | 15 | 0.36 | 129 | 16 | 0.34 | -0.10 | 0 |
| 0.8 | 114 | 10 | 0.31 | 113 | 13 | 0.26 | -0.38 | 0 |
| 0.85 | 98 | 4 | 0.32 | 92 | 10 | 0.15 | -0.84 | 0 |
| 0.9 | 74 | 3 | 0.20 | 64 | 5 | 0.08 | -1.76 | 0 |
| 0.95 | 48 | 1 | 0.12 | 42 | 1 | 0.10 | -4.52 | 0 |

**Supplementary Table 12.** Decision curve analysis for Crohn’s disease: net benefit of mid-end and high-end ultrasound models across threshold probabilities. This table summarizes the net benefit of mid-end and high-end intestinal ultrasound (IUS) models for predicting endoscopically active Crohn’s disease across clinically relevant threshold probabilities. The table includes comparisons with the theoretical "treat-all" and "treat-none" strategies, allowing evaluation of the incremental clinical utility of each ultrasound platform.

| **Threshold** | **TP_Mid** | **FP_Mid** | **NetBenefit_Mid** | **TP_High** | **FP_High** | **NetBenefit_High** | **NetBenefit_TreatAll** | **NetBenefit_TreatNone** |
| --- | --- | --- | --- | --- | --- | --- | --- | --- |
| 0.05 | 136 | 20 | 0.64 | 136 | 22 | 0.64 | 0.75 | 0 |
| 0.1 | 136 | 20 | 0.63 | 136 | 22 | 0.63 | 0.73 | 0 |
| 0.15 | 136 | 20 | 0.63 | 136 | 22 | 0.63 | 0.72 | 0 |
| 0.2 | 136 | 20 | 0.62 | 136 | 22 | 0.62 | 0.70 | 0 |
| 0.25 | 136 | 20 | 0.61 | 136 | 22 | 0.61 | 0.68 | 0 |
| 0.3 | 136 | 20 | 0.60 | 136 | 22 | 0.60 | 0.65 | 0 |
| 0.35 | 136 | 20 | 0.59 | 136 | 22 | 0.59 | 0.63 | 0 |
| 0.4 | 136 | 20 | 0.58 | 136 | 22 | 0.58 | 0.60 | 0 |
| 0.45 | 136 | 20 | 0.57 | 136 | 22 | 0.56 | 0.56 | 0 |
| 0.5 | 136 | 20 | 0.55 | 136 | 22 | 0.54 | 0.52 | 0 |
| 0.55 | 136 | 20 | 0.53 | 136 | 22 | 0.52 | 0.46 | 0 |
| 0.6 | 136 | 20 | 0.50 | 136 | 22 | 0.49 | 0.40 | 0 |
| 0.65 | 136 | 20 | 0.47 | 136 | 22 | 0.45 | 0.31 | 0 |
| 0.7 | 136 | 20 | 0.42 | 136 | 22 | 0.40 | 0.19 | 0 |
| 0.75 | 136 | 20 | 0.36 | 136 | 22 | 0.33 | 0.03 | 0 |
| 0.8 | 136 | 20 | 0.27 | 136 | 22 | 0.23 | -0.21 | 0 |
| 0.85 | 136 | 20 | 0.11 | 136 | 22 | 0.05 | -0.61 | 0 |
| 0.9 | 136 | 20 | -0.21 | 136 | 22 | -0.29 | -1.42 | 0 |
| 0.95 | 136 | 20 | -1.16 | 136 | 22 | -1.34 | -3.83 | 0 |

**References**

1. Pal P, Mateen MA, Pooja K, et al. Leveraging existing mid-end ultrasound machine for point-of-care intestinal ultrasound in low-resource settings: Prospective, real-world impact on clinical decision-making. Aliment Pharmacol Ther. 2024;60(5):633-647. [10.1111/apt.18155]

2. Pal P, Mateen MA, Sekaran A, et al. Can transperineal ultrasound replace endoscopic ultrasound in staging distal ulcerative colitis? Gut. 2025. [10.1136/gutjnl-2025-335681]

3. Novak KL, Nylund K, Maaser C, et al. Expert Consensus on Optimal Acquisition and Development of the International Bowel Ultrasound Segmental Activity Score [IBUS-SAS]: A Reliability and Inter-rater Variability Study on Intestinal Ultrasonography in Crohn’s Disease. Journal of Crohn's and Colitis. 2020;15(4):609-616. [10.1093/ecco-jcc/jjaa216]

4. Allocca M, Filippi E, Costantino A, et al. Milan ultrasound criteria are accurate in assessing disease activity in ulcerative colitis: external validation. United European Gastroenterol J. 2021;9(4):438-442. [10.1177/2050640620980203]

5. Allocca M, Jairath V, Sands BE, et al. International consensus on the use of intestinal ultrasound in inflammatory bowel disease trials. J Crohns Colitis. 2025;19(9). [10.1093/ecco-jcc/jjaf170]

6. Lu C, Rosentreter R, Parker CE, et al. International expert guidance for defining and monitoring small bowel strictures in Crohn's disease on intestinal ultrasound: a consensus statement. Lancet Gastroenterol Hepatol. 2024;9(12):1101-1110. [10.1016/s2468-1253(24)00265-6]
