# Supplementary material for: High-end intestinal ultrasound versus mid-end systems benchmarked against tandem ileocolonoscopy in inflammatory bowel disease (HUMID): a paired prospective, validating confirmatory study
Source: eClinicalMedicine. 2026 Apr 1;94:103856. doi: 10.1016/j.eclinm.2026.103856 (PMC13068867; doi:10.1016/j.eclinm.2026.103856)
Supplement: Protocol [file mmc2.docx]

**STUDY TITLE**

Comparing diagnostic accuracy of **H**igh-end intestinal **U**ltrasound versus **M**id-end ultrasound with tandem **I**leo-colonoscopy in inflammatory bowel **D**isease (the **HUMID** study): a paired, validating confirmatory study

**NAME OF THE INVESTIGATOR(S)**

**Dr. Partha Pal**

MD, DNB MRCP (UK), ESEGH, FASGE

Consultant Gastroenterologist, Asian Institute of Gastroenterology, Hyderabad

**CO-INVESTIGATOR(S)**

**Dr Mohammed Abdul Mateen**, DMRD, DNB

Chief, Diagnostic Radiology and Ultrasound, Asian Institute of Gastroenterology, Hyderabad

**SITE OF STUDY**

Asian Institute of Gastroenterology, Somajiguda, Hyderabad

1. **INTRODUCTION**

Inflammatory bowel disease (IBD), comprising Crohn’s disease (CD) and ulcerative colitis (UC), is a chronic, relapsing condition that requires accurate and timely diagnosis for effective management. The gold standard for assessing disease activity includes ileo-colonoscopy and cross-sectional imaging, both of which are costly and often inaccessible in resource-limited settings. Point-of-care ultrasound (POCUS) has emerged as a valuable, cost-effective alternative for managing IBD, particularly in low-resource environments.

Our previous research using a mid-end ultrasound machine (Siemens ACUSON S2000) demonstrated that POCUS significantly influenced clinical management, helping to avoid colonoscopy in more than half of the cases.[1] The study found that POCUS, when compared to ileo-colonoscopy, had a sensitivity of 80% and a specificity of 94.4% for CD, and a sensitivity of 80.8% and a specificity of 92.8% for UC. These results highlight the potential of POCUS to serve as an essential diagnostic tool in the management of IBD, particularly in settings where access to advanced diagnostic modalities is limited.[1]

While mid-end ultrasound machines have shown promise in managing IBD, there is a lack of comparative studies evaluating their diagnostic accuracy against high-end machines. High-end ultrasound devices, offer advanced imaging features such as extended field of view, speckle reduction, and enhanced computing power, which could potentially improve diagnostic accuracy. However, their high cost poses a significant barrier to widespread use, especially in resource-limited settings.

**2.0 RATIONALE**

The HUMID study addresses the critical need to evaluate the diagnostic accuracy of mid-end versus high-end intestinal ultrasound (IUS) machines in the context of inflammatory bowel disease (IBD) with ileocolonic involvement. In resource-limited settings, access to advanced diagnostic tools such as colonoscopy and cross-sectional imaging is often scarce, making point-of-care ultrasound (POCUS) an essential, cost-effective alternative for managing IBD.[2] While high-end ultrasound machines offer superior imaging quality through advanced features such as extended field of view, speckle reduction, and enhanced computing power, their high procurement costs limit their accessibility. Mid-end machines, equipped with high-frequency probes (≥5 MHz), provide a more affordable option, potentially enabling broader dissemination of POCUS in settings where resources are constrained. However, there is a perceived gap in the diagnostic precision of mid-end machines, as current literature on their efficacy remains limited. This study seeks to validate whether mid-end ultrasound machines can reliably match the diagnostic accuracy of high-end machines when compared to the gold standard of ileo-colonoscopy, ultimately aiming to inform resource allocation and optimize IBD management in diverse healthcare environments.

1. **STUDY OBJECTIVES**

**3a. Primary objective**

To compare the diagnostic accuracy of intestinal ultrasound (IUS) using a high-end ultrasound machine versus a mid-end ultrasound machine in relation to ileo-colonoscopy for assessing ileocolonic involvement in IBD.

**Secondary Objectives:**

To evaluate changes in clinical management decisions following IUS with a high-end ultrasound machine compared to a mid-end machine.

**4.0 STUDY POPULATIONS**

**Inclusion Criteria:** Adult patients (18-75 years) with a confirmed diagnosis of IBD (CD or UC) involving colon and/or terminal ileum who are attending outpatient or inpatient care at the Asian Institute of Gastroenterology.

**Exclusion Criteria:** Patients with known contraindications to colonoscopy, pregnant women, and those unable to provide informed consent.

**5.0 DESIGN AND DURATION OF THE STUDY**

Cross-sectional study, follow up until ileo-colonoscopy (same or next day)

Duration of study (Recruitment period) : 6-12 months (estimated sample size 100)

**6.0 METHODOLOGY**

The HUMID (High-end vs. Mid-end Ultrasound in IBD Diagnostics) study is a prospective, paired, validating confirmatory study aimed at comparing the diagnostic accuracy of mid-end versus high-end intestinal ultrasound (IUS) machines in patients with inflammatory bowel disease (IBD) involving the ileocolonic region. The study specifically contrasts the performance of the mid-end Siemens ACUSON S2000 and the high-end Samsung RS85 ultrasound machines, using ileo-colonoscopy as the reference standard.

Upon enrollment, each patient will undergo a clinical assessment by an IBD specialist using the Harvey–Bradshaw Index (HBI) for Crohn’s Disease (CD) and the Simple Clinical Colitis Activity Index (SCCAI) for Ulcerative Colitis (UC). Patients will be categorized into three groups: flare/relapse, response to new therapy, and clinically quiescent disease. Point-of-care ultrasound (POCUS) will be performed using both the Siemens ACUSON S2000 (mid-end) and Samsung RS85 (high-end) machines, with patients asked to fast prior to the examination, though this is not mandatory. The Siemens ACUSON S2000 will utilize a high-frequency linear probe (9 L4, 4–9 MHz) and will use pediatric abdomen and thyroid presets with a trapezoid view for bowel visualization. The Samsung RS85 will employ linear 2-10 MHz and 2-18 MHz probes, offering advanced imaging features such as extended field of view and speckle reduction. Both ultrasound examinations will be conducted jointly by a GI sonologist and an IBD specialist, with findings documented by a research associate and consensus reached by the examiners. Ileo-colonoscopy will be performed on the same or following day if clinically indicated, providing the diagnostic gold standard against which the accuracy of both ultrasound machines will be assessed.

**7.0 OUTCOME MEASURES**

- Diagnostic accuracy (sensitivity, specificity, positive predictive value, and negative predictive value) of mid-end versus high-end ultrasound machines in detecting ileocolonic involvement in IBD.
- Proportion of patients where clinical management was altered based on IUS findings.

**8.0 SAMPLE SIZE CALCULATION**

This is a pilot study and hence we plan to recruit 100 patients to have a reasonable sample size for achieving clinical significance

**9.0** **STATISTICAL ANALYSIS**

Data shall be analyzed to compare the sensitivity, specificity, positive predictive value (PPV), and negative predictive value (NPV) of the mid-end versus high-end ultrasound machines. Paired comparisons between the two machines and against the reference standard (ileo-colonoscopy) shall be conducted using appropriate statistical tests. Statistical Package for the Social Sciences version 26 (Armonk, NY: IBM Corp.) shall be used for statistical analysis and p value <0.05 shall be considered significant.

**10.0 ETHICAL JUSTIFICATION OF THE STUDY**

The study titled "Comparing Diagnostic Accuracy of High-End Intestinal Ultrasound Versus Mid-End Ultrasound with Tandem Ileo-Colonoscopy in Inflammatory Bowel Disease (the HUMID Study)" is designed to evaluate the diagnostic accuracy of high-end versus mid-end intestinal ultrasound machines in the context of inflammatory bowel disease (IBD) with ileocolonic involvement. This study aims to address the critical need for effective, cost-efficient diagnostic tools in resource-limited settings.

1. **Patient Benefit and Risk Reduction:**
   - **Non-Invasive Assessment:** The use of both mid-end and high-end ultrasound machines for evaluating bowel conditions provides a non-invasive approach to diagnosis, enhancing patient comfort and safety. This reduces the need for more invasive procedures such as ileo-colonoscopy, which carries risks including bleeding, perforation, and patient discomfort.
   - **Improved Diagnostic Accuracy:** By comparing the diagnostic accuracy of mid-end and high-end ultrasound machines against the gold standard of ileo-colonoscopy, the study aims to identify the most effective and cost-efficient tool for managing IBD. This could lead to better disease management and outcomes for patients.
2. **Advancing Medical Knowledge:**
   - **Innovative Evaluation:** The study explores the comparative efficacy of different ultrasound machines, contributing valuable insights into the utility of mid-end versus high-end devices in clinical practice. This could improve diagnostic protocols and resource allocation in settings with limited access to advanced imaging.
   - **Enhanced Disease Management:** Findings from the study could potentially revolutionize the management of IBD, providing a more accessible diagnostic option that can be widely implemented in resource-constrained environments.
3. **Ethical Study Design:**
   - **Informed Consent:** Participants will undergo a thorough informed consent process, ensuring they are fully informed about the study’s procedures, risks, and benefits. This process ensures that patients can make well-informed decisions regarding their participation.
   - **Minimizing Risk:** The study design prioritizes minimal risk to participants. The use of non-invasive ultrasound techniques, combined with ileo-colonoscopy only when clinically necessary, ensures that unnecessary exposure to invasive procedures is minimized.
   - **Confidentiality:** Patient confidentiality will be rigorously protected. All data collected will be anonymized and securely stored, ensuring privacy and ethical handling of patient information.
4. **Clinical Relevance and Utility:**
   - **Direct Clinical Impact:** The study’s outcomes will have immediate clinical applications. If the mid-end ultrasound proves to be as effective as the high-end model, it could facilitate broader adoption of ultrasound technology in settings with limited resources, thus improving patient care and diagnostic efficiency.
5. **Multidisciplinary Approach:**
   - **Comprehensive Evaluation:** The study involves a multidisciplinary team of gastroenterologists, ultrasound specialists, and researchers, ensuring a thorough and objective evaluation of the imaging techniques.

In summary, the HUMID study is ethically justified as it seeks to enhance patient care by validating a cost-effective diagnostic tool, potentially reducing the reliance on invasive procedures, and improving the management of inflammatory bowel disease. The study’s design incorporates thorough informed consent, minimizes risk, and maintains confidentiality, further supporting its ethical integrity.

**Reference**

1. Pal P, Mateen MA, Pooja K, Marri UK, Gupta R, Tandan M, Reddy DN. Leveraging existing mid-end ultrasound machine for point-of-care intestinal ultrasound in low-resource settings: Prospective, real-world impact on clinical decision-making. Aliment Pharmacol Ther. 2024 Sep;60(5):633-647. doi: 10.1111/apt.18155. Epub 2024 Jul 8. PMID: 38975815.
2. Allocca M, Fiorino G, Bonifacio C, Furfaro F, Gilardi D, Argollo M, Peyrin-Biroulet L, Danese S. Comparative Accuracy of Bowel Ultrasound Versus Magnetic Resonance Enterography in Combination With Colonoscopy in Assessing Crohn's Disease and Guiding Clinical Decision-making. J Crohns Colitis. 2018 Nov 15;12(11):1280-1287. doi: 10.1093/ecco-jcc/jjy093. PMID: 29982361.
